# Identification of a Contact-Dependent Growth Inhibition (CDI) System That Reduces Biofilm Formation and Host Cell Adhesion of *Acinetobacter baumannii* DSM30011 Strain

**DOI:** 10.3389/fmicb.2019.02450

**Published:** 2019-10-30

**Authors:** Morgane Roussin, Sedera Rabarioelina, Laurence Cluzeau, Julien Cayron, Christian Lesterlin, Suzana P. Salcedo, Sarah Bigot

**Affiliations:** ^1^Cell Biology of Bacterial Pathogenicity Team, Laboratory of Molecular Microbiology and Structural Biochemistry, CNRS UMR 5086, University of Lyon, Lyon, France; ^2^Cell to Cell DNA Transfer Team, Laboratory of Molecular Microbiology and Structural Biochemistry, CNRS UMR 5086, University of Lyon, Lyon, France

**Keywords:** type V secretion system, contact-dependent growth inhibition, *Acinectobacter baumannii*, biofilm, cell adhesion ability

## Abstract

*Acinetobacter baumannii* is a multidrug-resistant nosocomial opportunistic pathogen that is becoming a major health threat worldwide. In this study, we have focused on the *A. baumannii* DSM30011 strain, an environmental isolate that retains many virulence-associated traits. We found that its genome contains two loci encoding for contact-dependent growth inhibition (CDI) systems. These systems serve to kill or inhibit the growth of non-sibling bacteria by delivering toxins into the cytoplasm of target cells, thereby conferring the host strain a significant competitive advantage. We show that one of the two toxins functions as a DNA-damaging enzyme, capable of inducing DNA double-stranded breaks to the chromosome of *Escherichia coli* strain. The second toxin has unknown catalytic activity but stops the growth of *E. coli* without bactericidal effect. In our conditions, only one of the CDI systems was highly expressed in the *A. baumannii* DSM30011 strain and was found to mediate interbacterial competition. Surprisingly, the absence of this CDI system promotes adhesion of *A. baumannii* DSM30011 to both abiotic and biotic surfaces, a phenotype that differs from previously described CDI systems. Our results suggest that a specific regulation mediated by this *A. baumannii* DSM30011 CDI system may result in changes in bacterial physiology that repress host cell adhesion and biofilm formation.

## Introduction

In Gram-negative bacteria, the two-partner secretion (TPS) pathway, also known as type Vb secretion system (T5bSS), mediates the translocation across the outer membrane of large, mostly virulence-related, TpsA proteins (Guérin et al., 2017). Functions of the TpsA secreted through the TPS pathway are diverse ranging from cytolysis, adhesion, and iron acquisition to contact-dependent growth inhibition (CDI) (van Ulsen et al., 2014). CDI system was the first secretion system identified to deliver toxin into neighboring cells, arming bacteria with a killing mechanism for outcompeting non-kin cells and establishment of self-communities (Aoki et al., 2005). Growth inhibition involves direct physical contact between bacteria and depends on the production of toxin–antitoxin pairs (Willett et al., 2015). This mechanism exploits the CdiA/CdiB subfamily of TPS systems to export CdiA to the surface through the cognate CdiB transporter and deliver into the cytosol of the target bacterium the last ∼300 C-terminal toxic residues of the CdiA proteins, called CdiA-CT. The C-terminal domain is delimited by conserved motifs of unknown function such as (Q/E)LYN in *Burkholderia*, VENN in most bacteria, or yet other motifs in *Pseudomonas* (Aoki et al., 2005; Anderson et al., 2012; Mercy et al., 2016). The presence of the cytoplasmic immunity protein CdiI protects CDI^+^ bacteria by interacting with the cognate CdiA-CT toxin and neutralizing its toxic activity. CdiA-CT is highly variable and shows various folds and activities (tRNase, DNase, and pore forming), allowing for a wide diversity of distinct toxins to be deployed to target bacteria (Zhang et al., 2011).

Contact-dependent growth inhibition systems are widespread among Gram-negative bacteria, as *cdi* gene clusters are found in several α-, β-, and γ-proteobacteria. They have been extensively studied in Enterobacteria and *Burkholderia* species, and recent work investigated their role in *Acinetobacter* species (Harding et al., 2017). *Acinetobacter baumannii* can be found associated with severe infections in humans, exhibiting multidrug resistance and causing fatal infections in susceptible hosts, such as patients in intensive care units. *A. baumannii* resists desiccation and forms biofilms that may contribute to its persistence in the clinical devices, causing acute infections. The molecular mechanisms implicated in infection by *A. baumannii* and the virulence factors associated with this process are still unclear. Recent studies investigated the potential implication of TPS systems in *A. baumannii* pathogenesis. The TpsA proteins characterized in strains *A. baumannii* ATCC 19606(T) and clinical AbH12O-A2 are both adhesins that mediate adherence to eukaryotic cells (Darvish Alipour Astaneh et al., 2014; Pérez et al., 2016), and TpsA of *A. baumannii* AbH12O-A2 was shown to contribute to virulence in models of mouse systemic infection and *Caenorhabditis elegans* (Pérez et al., 2016). Interestingly, our *in silico* analysis revealed that these two adhesins associated with their respective CdiB and CdiI partners constitute putative CDI systems, suggesting a potential involvement of these systems in the virulence of *A. baumannii*. This is in line with studies in other organisms suggesting a role for CDI systems beyond bacterial competition. Indeed, several CdiA promotes bacterial auto-aggregation and biofilm formation in *Escherichia coli*, *Pseudomonas aeruginosa*, and *Burkholderia thailandensis* (Garcia et al., 2013; Ruhe et al., 2015; Mercy et al., 2016), as well as intracellular escape and immune evasion of *Neisseria meningitidis* (Talà et al., 2008), functions that are required for the virulence of several pathogens (Gallagher and Manoil, 2001; Rojas et al., 2002; Guilhabert and Kirkpatrick, 2005; Gottig et al., 2009). Recently, *in silico* analysis revealed the identification of more than 40 different CDI systems in pathogenic *Acinetobacter* genomes that have been sorted into type I and II groups (De Gregorio et al., 2019). While sequencing the genome from *A. baumannii* DSM30011 strain (Repizo et al., 2017), we have also identified two *cdiBAI* loci potentially encoding type I and II CDI systems. *A. baumannii* DSM30011, an environmental strain isolated in 1944 from resin-producing guayule plants, has many of the characteristics of clinical strains and was shown to use a type 6 secretion system (T6SS) for bacterial competition and colonization in the model organism *Galleria mellonella* (Repizo et al., 2015). In this study, we used live-cell microscopy to characterize the function of CdiA-CT toxins when produced in *E. coli* cells. Using transcriptional fusions, we show that only one CDI system is expressed in *A. baumannii* DSM30011 and promotes interbacterial competition but is surprisingly a limiting factor for the adhesion process.

## Results

### The *Acinetobacter baumannii* DSM30011 Genome Contains Two Predicted CDI Systems

**FIGURE 1:**
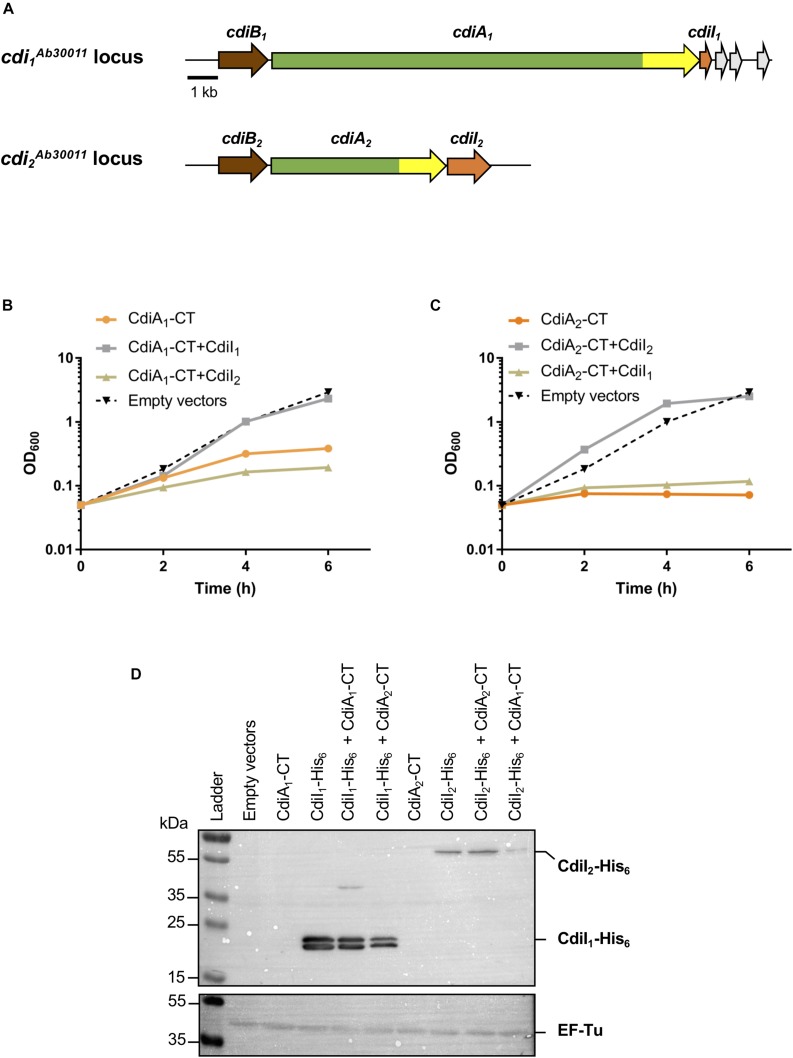
The production of CT domains of CdiA_1_ or CdiA_2_ in *Escherichia coli* is toxic, and *cdiI* genes encode immunity proteins. **(A)** Schematic of the *cdi_1_^Ab30011^* and *cdi_2_^Ab30011^* loci. Genes encoding putative CdiB transporters, CdiA exoproteins, and CdiI immunity proteins are colored, respectively, in brown, green, and orange. Gray arrows represent putative orphan immunity genes. The highly variable nucleotide regions encoding CdiA-CT domains are highlighted in yellow. Black bar represents the 1-kb scale. Based on sequenced *Acinetobacter baumannii* DSM30011 genome (Repizo et al., 2017), CdiB_1_, CdiA_1_, and CdiI_1_ correspond, respectively, to PNH15603.1, PNH15604.1, and PNH15605.1 proteins and CdiB_2_, CdiA_2_, and CdiI_2_ to PNH14818.1, PNH14817.1, and PNH14816.1 proteins. **(B,C)** Effect of CdiA_1_-CT **(B)** or CdiA_2_-CT **(C)** production in *E. coli* DH5α strain. CdiA-CT production was induced at 0 min with or without CdiI, and growth was monitored by measuring the optical density at 600 nm (OD_600_). **(D)** After 6 h of culture, *E. coli* cell extracts containing an indicated set of produced proteins were analyzed to probe the production of CdiI-His_6_ and the cytoplasmic EF-Tu control using the anti-pentaHis (top panel) and anti-EF-Tu monoclonal antibodies (bottom panel). Molecular weight marker (kDa) is indicated on the left.

In the course of this study, we performed a bioinformatic search to obtain the global repartition and representation of TPS systems among *A. baumannii* species. Each subset of TpsA was used to blast against the *A. baumannii* sequence database. Based on their sequence, TpsA proteins can be phylogenetically classified into at least five subfamilies with distinct functions: (i) the CDI CdiA proteins (Aoki et al., 2005), (ii) the hemolysins/cytolysins such as ShlA of *Serratia marcescens* (Braun et al., 1992), (iii) the adhesins such as filamentous hemagglutinin (FHA) of *Bordetella pertussis* (Relman et al., 1989), (iv) HxuA-type proteins involved in iron acquisition (Fournier et al., 2011), and (v) TpsA with unknown specific activities (Faure et al., 2014). The blast search revealed that the CDI system subfamily is predominantly represented within *A. baumannii* strains with the exception of some genomes comprising Hxu system homologues. We detected two loci in our *A. baumannii* DSM30011 laboratory model strain containing gene organization related to the “*Escherichia coli*”-type CDI systems (Figure 1A). We renamed them as *cdi_1_^Ab30011^* (encoding proteins PNH15603.1, PNH15604.1, and PNH15605.1) and *cdi_2_^Ab30011^* (encoding proteins PNH14818.1, PNH14817.1, and PNH14816.1). The CdiA proteins of these systems share only 9.6% identity overall, but both contain the highly conserved VENN motif (PF04829) that delimits the N- and C-terminal (CT) domains. The sequence analysis of CdiA_1_ revealed that the N-terminal domain of this very large protein (532 kDa) harbors long stretches of imperfect repeats predicted to form β-helix folds, that is, β-strand structure organized in fibrous (Kajava et al., 2001), which classifies it as a type II CdiA protein (De Gregorio et al., 2019). CdiA_1_-CT does not contain any conserved domain. The smaller CdiA_2_ protein (204 kDa) belongs to the type I CdiA and its CdiA_2_-CT preceded by the small-helical DUF637 domain found in many CdiA proteins (Iyer et al., 2011) contains a Tox-REase7 nuclease domain. Three potential orphan *cdiI* genes encoding the PNH15606.1, PNH15607.1, and PNH15608.1 proteins are located downstream of the *cdi*_1_ locus. Indeed, PNH15606.1 and PNH15607.1 proteins contain an Imm23 domain, and PNH15608.1 protein has a Smi1/Knr4 superfamily domain typically found in immunity proteins present in bacterial polymorphic toxin systems (Iyer et al., 2011; Zhang et al., 2011).

### CdiA_1_-CT/CdiI_1_ and CdiA_2_-CT/CdiI_2_ Are Two Non-interchangeable Toxin–Antitoxin Pairs

To address the toxicity of CT domains, we generated pBAD33 plasmid derivatives producing each CdiA-CT (from the VENN motif to the stop codon) in the presence of arabinose. To assess the CdiI immunity property, nucleotide sequences encoding CdiI fused to 6xHis tag were introduced in pTrc99a plasmid and induced with isopropyl-β-D-1-thiogalactopyranoside (IPTG). The production of CdiA_1_-CT alone stops the growth of the *E. coli* DH5α strain after 4 h of induction (Figure 1B). Unlike CdiA_1_-CT, CdiA_2_-CT is highly toxic in *E. coli* where its induction inhibits the growth (Figure 1C). Cells coproducing CdiI_1_ and CdiA_1_-CT or CdiI_2_ and CdiA_2_-CT are protected from the toxic effect of the toxins and exhibited growth equivalent to that of cells containing the empty vector (Figures 1B,C). In contrast, the production of CdiI_1_ or CdiI_2_ with CdiA_2_-CT or CdiA_1_-CT, respectively, does not suppress toxicity. Both CdiI-6xHis were detected in *E. coli* cells in the presence or absence of CdiA-CT using Western blot experiment (Figure 1D) showing that the inability to rescue the growth defect caused by a non-cognate CdiA-CT is not due to a lack of CdiI production. CdiA_1_-CT/CdiI_1_ and CdiA_2_-CT/CdiI_2_ therefore function as pairs of toxin–antitoxin, and these systems are not interchangeable.

### CdiA_2_-CT^Ab30011^ Toxin Induces DNA Damage in *E. coli*

**FIGURE 2:**
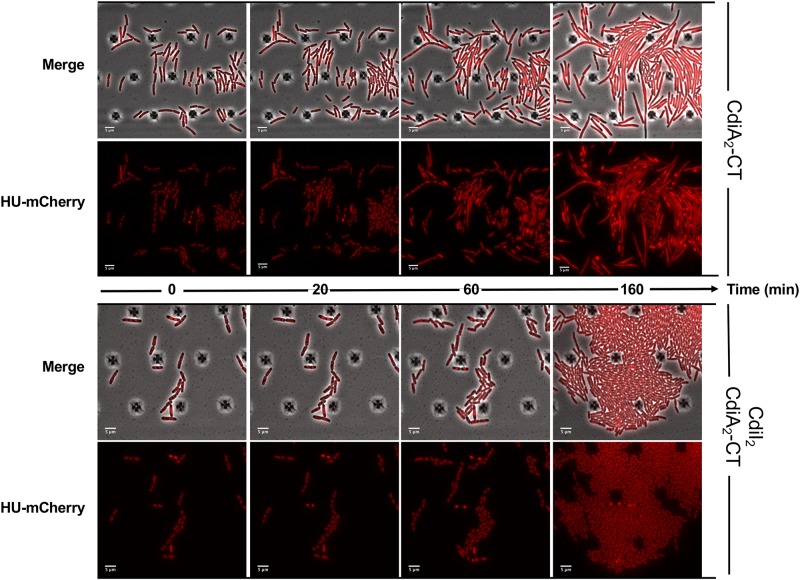
The production of CdiA_2_-CT in *Escherichia coli* disorganizes the nucleoid localization. Time-lapse fluorescence microscopy of the recombinant HU-mCherry protein produced by *E. coli* MG1655 strain after induction of the CdiA_2_-CT in the absence or in presence of its cognate CdiI_2_ immunity protein. The scale bar corresponds to 5 μm.

**FIGURE 3:**
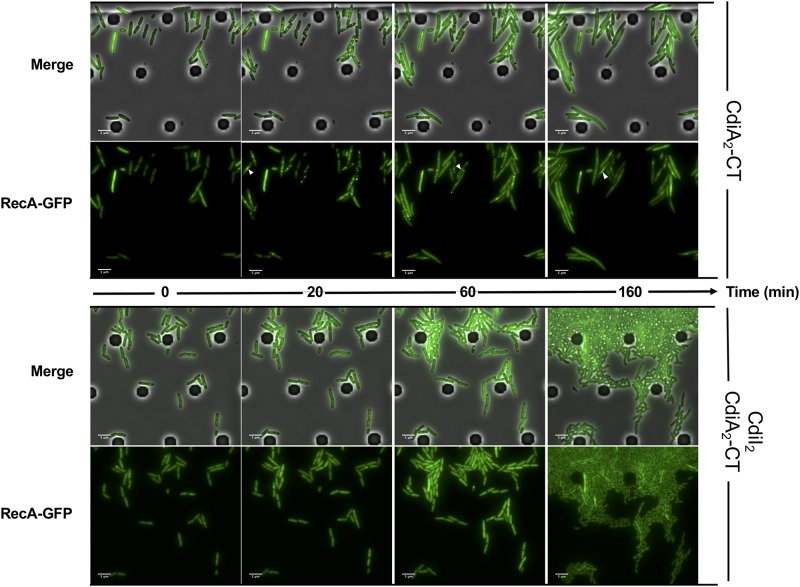
The production of CdiA_2_-CT in *Escherichia coli* induces DNA damages. Time-lapse fluorescence microscopy of the recombinant RecA-GFP protein produced by *E. coli* MG1655 strain after induction of the CdiA_2_-CT in the absence or in the presence of its cognate CdiI_2_ immunity protein. White arrows indicate examples of bundles of RecA-GFP. The scale bar corresponds to 5 μm. GFP, green fluorescent protein.

CdiA_2_-CT contains a restriction endonuclease-like domain belonging to the Tox-REase7 family (Pfam PF15649) mostly found in CdiA of *Pseudomonas* and *Acinetobacter* species (Zhang et al., 2012; Mercy et al., 2016). To determine whether CdiA_2_-CT displays nuclease activity when expressed in *E. coli* cells, we performed real-time microscopy visualization of the nucleoid-associated HU protein after induction of the toxin. HU is a widely conserved histone-like protein very abundant in the bacterial cytoplasm that binds to DNA in a non-specific manner. Owing to its nucleoid association, HU localization reveals the global organization of the chromosome and potential alterations. We grew a CdiA_2_-CT-producing *E. coli* MG1655 strain in which the *hupA* gene encoding the α-subunit of HU is fused to the *mcherry* gene at the endogenous locus. Twenty minutes after CdiA_2_-CT induction, the majority of the cells contain organized and well-segregated nucleoids (Figure 2 and Supplementary Movie S1). However, after 60 min, the nucleoids condense as a dense mass at midcell of the bacteria or show a diffused localization pattern. This global nucleoid disorganization is followed by cell filamentation indicative of cell division inhibition. These chromosome alterations are associated with cell death, as CdiA_2_-CT-producing cells are not able to form viable colony units (Supplementary Figure S1A). No filamentation is observed after induction of the CdiA_2_-CT in *recA*- cells showing that cell division arrest depends on the homologous recombination RecA protein (Supplementary Figure S1B). In contrast to bacteria producing only CdiA_2_, cells coproducing the CdiI_2_ immunity protein suffer no loss of viability and retain normal chromosome organization and cell division (Figure 2, Supplementary Movie S2, and Supplementary Figure S1A). The perturbation of DNA organization together with the RecA-dependent filamentation observed in the presence of CdiA_2_-CT suggests that this toxin induces DNA damage to the chromosomal DNA. To test this hypothesis, we examined the localization pattern of the RecA protein, which has been reported to polymerize into large intracellular structures in response to DNA lesions (Lesterlin et al., 2014). To do so, we used an *E. coli* MG1655 strain expressing a carboxy-terminal fusion of RecA to green fluorescent protein (GFP) and wild-type (wt) RecA, both expressed from wt chromosome *recA* promoters. Before induction of CdiA_2_-CT, RecA-GFP fluorescence appears uniformly distributed in most cells (Figure 3; *t* = 0 h and Supplementary Movie S3). From 20 min after production of CdiA_2_-CT, fluorescent RecA-GFP structures form in the majority of cells, and we observed the formation of RecA bundles reported to promote homologous recombination repair between distant regions of homology (Lesterlin et al., 2014). As expected, RecA-GFP fluorescence is diffuse when CdiA_2_-CT is co-expressed with CdiI_2_, reflecting the absence of DNA damage (Figure 3 and Supplementary Movie S4). Altogether, these results demonstrate that CdiA_2_-CT creates multiple DNA breaks that cannot be repaired by the cells leading to growth inhibition, loss of chromosome organization, and eventual cell death.

### CdiA_1_-CT Toxin Inhibits the Growth of *E. coli*

**FIGURE 4:**
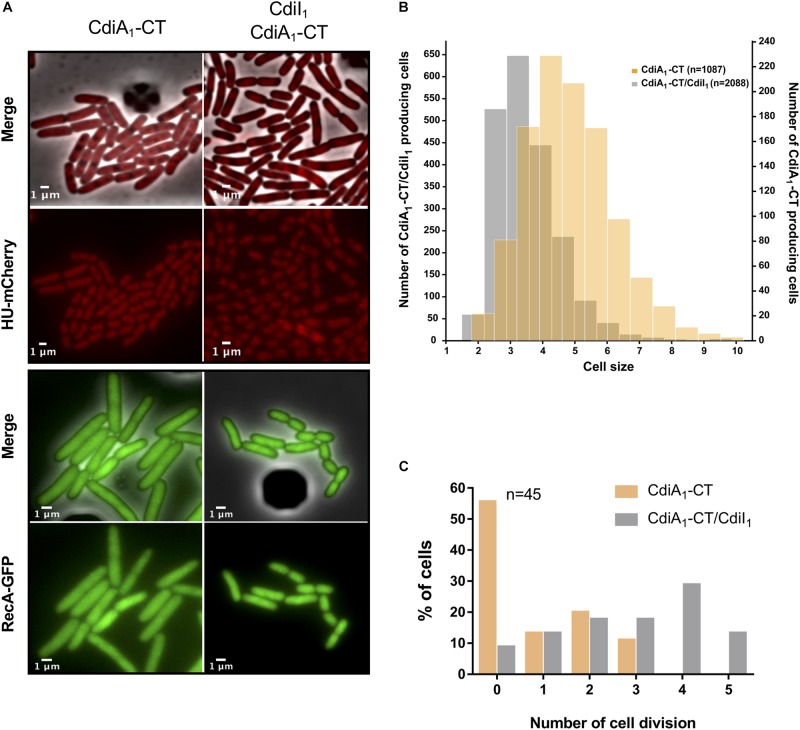
The CdiA_1_-CT toxin stops the growth of *Escherichia coli*. **(A)** Fluorescence microscopy of recombinant HU-mCherry or RecA-GFP produced by *E. coli* MG1655 strains 130 min after induction of CdiA_1_-CT in the absence or in the presence of its cognate CdiI_1_ immunity protein. The scale bar is indicated on the lower left. **(B)** Cell length analysis of RecA-GFP producing *E. coli* MG1655 strains 3 h after induction of CdiA_1_-CT in the absence or in the presence of its cognate CdiI_1_ immunity protein. *n* indicates the number of analyzed cells. **(C)** HU-mCherry producing *E. coli* MG1655 strains were grown in M9-casa after induction of CdiA_1_-CT with or without CdiI_1_, and real-time microscopy was performed in order to calculate the number of division over 45 single bacteria. GFP, green fluorescent protein.

In order to get insight into the mechanism of growth inhibition generated by the production of CdiA_1_-CT in *E. coli* (Figure 1B), we analyzed in real-time microscopy the localization pattern of the recombinant HU-mCherry and RecA-GFP proteins after induction of CdiA_1_-CT in the presence or absence of its cognate CdiI_1_ immunity protein. Microscopy analysis showed no diffusion of the HU-mCherry in CdiA_1_-CT-producing cells and RecA-GFP formed no spot or bundle, indicating that the toxin does not induce nucleoid disruption nor DNA damage (Figure 4A and Supplementary Movies S5–S8). However, we noticed CdiA_1_-CT production led to increased cell size (Figure 4B) and division arrest in more than half of the cell population (Figure 4C). These results are consistent with the observed cell viability decrease 2 h post production of the CdiA_1_-CT followed by a plateau of the number of colony-forming unit (CFU)/ml indicating a cell growth defect (Supplementary Figure S2A). To assess if these arrested cells are alive, we used the Live/Dead assay on the basis of the green fluorescent SYTO9 entering all cells to stain nucleic acid and the propidium iodide entering only dead cells with damaged cytoplasmic membranes. Our results showed the CdiA_1_-CT-producing cells are not dying once they have stopped growing and that the percentage of cell death was similar to that of cells producing CdiA_1_-CT in the presence of CdiI_1_ (Supplementary Figure S2B).

### Unlike the *cdiBAI*_2_ Locus, the *cdiBAI*_1_ Genes Are Expressed in *A. baumannii* DSM30011

**FIGURE 5:**
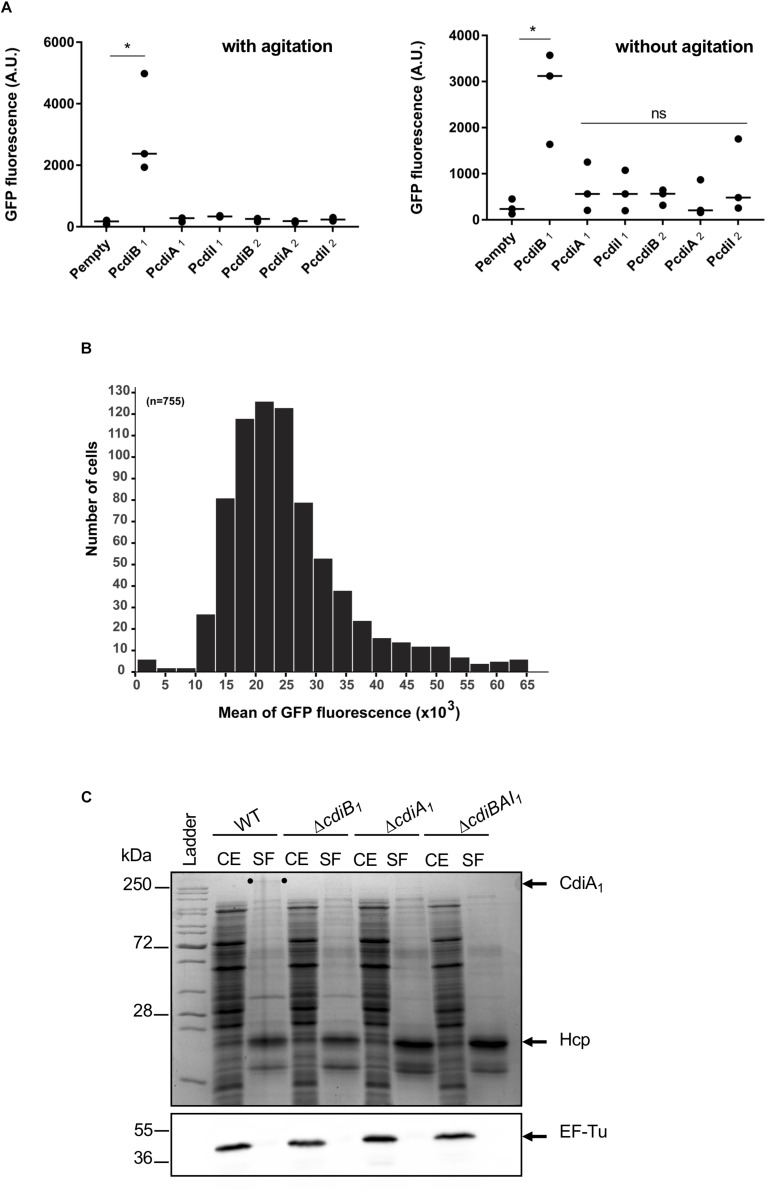
Only the *cdiBAI*_1_ genes are expressed in *Acinetobacter baumannii* DSM3011. **(A)** GFP fluorescence measurements for potential promoter regions of *cdi* genes. A strain harboring plasmid with a *gfp* reporter gene without a promoter (Pempty) served as a negative control. For statistical analyses, a non-parametric one-way ANOVA with Dunn’s multiple comparison test was performed. *^∗^p* = 0.0184 (with agitation) and *p* = 0.028 (without agitation); ns: not significant. **(B)** Repartition of the GFP fluorescence intensities within a sibling population harboring the P*cdiB*_1_ fusion. *n* = number of analyzed cells. **(C)** Cell extract (CE) and secreted fraction (SF) of wild-type and mutant strains grown overnight were analyzed by Coomassie blue staining and Western blot analysis using anti-EF-Tu antibody. The migration position of CdiA_1_ protein is indicated by two dots. Molecular marker (kDa) is indicated on the left. GFP, green fluorescent protein.

Most of the *cdi* genes are not expressed in laboratory growth conditions (Cope et al., 1994; Rojas et al., 2002; Mercy et al., 2016). In order to get insight into the expression profile of *cdi* genes present in *A. baumannii* DSM30011, we constructed plasmids containing transcriptional fusions between DNA region upstream of these genes and the *gfp* reporter. *A. baumannii* DSM30011 reporter strains were grown in liquid Luria–Bertani (LB)-rich medium at 37°C with agitation and static conditions, and the GFP fluorescence intensity was measured at 6 and 8 h, respectively. Equivalent results were obtained for each growth condition (Figure 5A). The level of GFP fluorescence of P*cdiB*_2_, P*cdiA*_2_, and P*cdiI*_2_ fusions is identical to that of the promoterless fusion, indicating that DNA regions upstream of these genes do not have any promoter activity and that the *cdi*_2_ locus might not be expressed under the growth conditions tested (Figure 5A). In contrast, the P*cdiB*_1_ fusion produces a really high level of GFP compared to the regions upstream the *cdiA*_1_ and *cdiI*_1_ genes that do not exhibit any promoter activity (Figure 5A). In addition, the GFP fluorescence is quite homogeneous within the population, as all individual bacteria produce high levels of GFP fluorescence (Figure 5B). In order to determine whether CdiA_1_ is produced in the tested conditions, we tried to detect this protein using sodium dodecyl sulfate polyacrylamide gel electrophoresis (SDS-PAGE). Overnight cultures were fractionated to analyze the protein profile of the whole cell lysate and secreted fraction. A band with a slower mobility than 250-kDa molecular weight marker was detected in the secreted fraction of the wt strain, which was identified as CdiA_1_ protein by mass spectrometry (Figure 5C). For controls, we confirmed that this band disappeared in the Δ*cdiA*_1_ mutant and that the CdiA_1_ transport required CdiB_1_ because no CdiA_1_ is secreted in a Δ*cdiB*_1_ mutant probably owing to the instability of the protein that is trapped in the periplasm, which is consistent with previous reports on FHA undergoing a rapid proteolytic degradation in the periplasm in the absence of its transporter FhaC (Jacob-Dubuisson et al., 1997; Guédin et al., 1998). Furthermore, the secretion of CdiA_1_ is not due to cell lysis, as the cytoplasmic EF-Tu protein is only found in the whole cell fraction and Hcp, identified by mass spectrometry, the main component of the T6SS, is detected in the secreted fraction (Figure 5C). Knowing that CdiA_1_ is highly produced and can be visualized by Coomassie blue staining, this suggests that the three *cdiBAI*_1_ genes are transcribed as a polycistronic mRNA from the same promoter upstream *cdiB*_1_.

### The *cdiBAI*_1_ Locus Mediates Bacterial Competition

**FIGURE 6:**
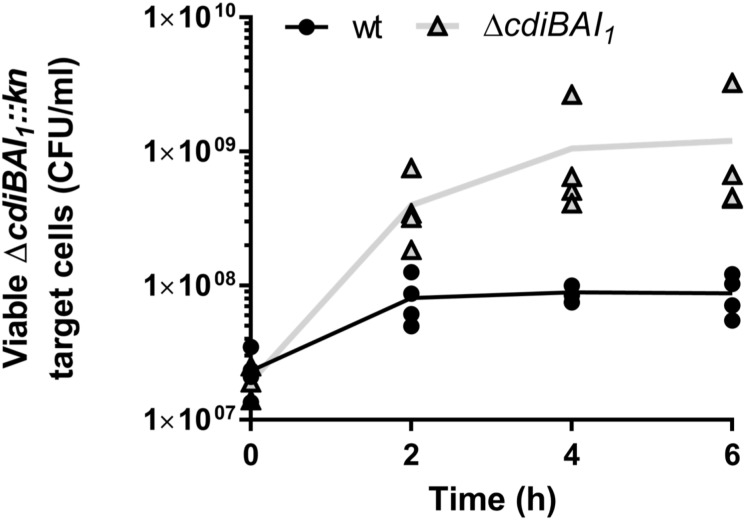
Growth inhibition mediated by the CDI_1_ system of strain *Acinetobacter baumannii* DSM30011. Both attacker and target strains were grown in LB separately to OD_600_ = 0.35 and mixed to a 4:1 (attacker:target) ratio. At various times of co-incubation, the *cdiBAI_1_:kn* target was spread on LB agar supplemented with kanamycin to measure the colony-forming unit (CFU)/ml. For statistical analyses, a non-parametric Mann–Whitney test was performed. ^∗^*p* = 0.0286 at 4 and 6 h. GFP, green fluorescent protein; LB, Luria–Bertani.

As only the CDI_1_ system is turned on under the tested conditions and we have not yet determined the regulatory pathway of the *cdi*_2_ locus, we pursued in *A. baumannii* the characterization of the *cdiBAI*_1_ system. To determine whether this system functions as a CDI system, the entire locus was replaced by a kanamycin cassette or deleted generating the Δ*cdiBAI_1_:kn* and Δ*cdiBAI*_1_ mutants. Next, we performed competition experiments by mixing the wt or Δ*cdiBAI*_1_ attacker strains with the Δ*cdiBAI_1_:kn* target strain and measure the CFU/ml of the target over time. As seen in Figure 6, wt strain inhibits the growth of the isogenic Δ*cdiBAI_1_:kn* target strain by ∼1 log after 6 h of growth competition, whereas no growth inhibition is observed with the Δ*cdiBAI*_1_ attacker strain. No significant viability defect between the wt and mutant strains was observed, indicating that the ability of wt strain to outcompete the Δ*cdiBAI*_1_ is not due to a growth rate difference (Supplementary Figure S3).

### *cdi*_1_ Locus Decreases *A. baumannii* DSM30011 Biofilm Formation and Adhesion to Epithelial Cells

**FIGURE 7:**
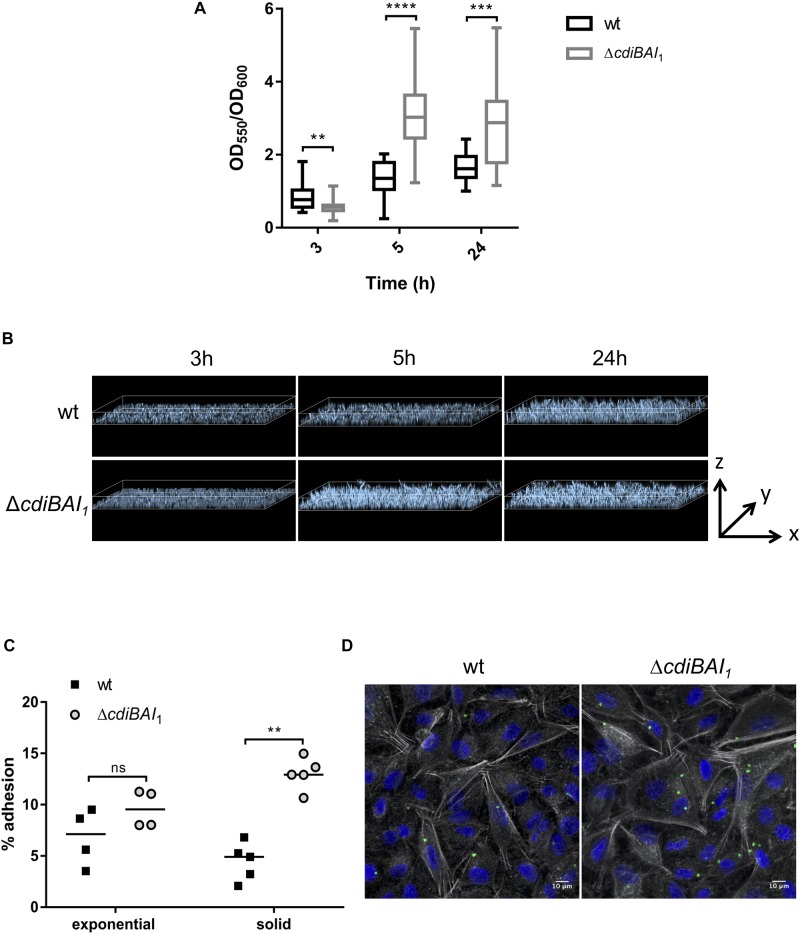
The adhesive properties of *Acinetobacter baumannii* DSM30011 is enhanced in the absence of *cdiBAI*_1_ locus. **(A)** Biofilm formation assayed by crystal violet staining. *A. baumannii* DSM30011 wild-type (wt) and Δ*cdiBAI*_1_ strains were statically grown at 37°C for 3, 5, and 24 h. The biofilm biomass was estimated by calculating the OD_550_/OD_600_ ratio from three independent experimental replicates. For statistical analyses, a non-parametric Mann–Whitney test was performed. ^∗∗^*p* = 0.0045, ^∗∗∗^*p* = 0.0004, ^∗∗∗∗^*p* < 0.0001. **(B)** Confocal microscopy of biofilm formed by the wild-type and Δ*cdiBAI*_1_ strains grown on glass-bottom slides in static conditions. Images correspond to the depth analysis of a 3D reconstruction of z-stacks obtained for the different strains at 3, 5, and 24 h. The height of the *z* axis is between 0 and 10 μm. Bacteria were labeled with DAPI. **(C)** Adhesion assay in which A549 cells were infected with *A. baumannii* DSM30011 wt or Δ*cdiBAI*_1_ strains at a MOI of 100 for 2 h. Data are expressed as a percentage of *A. baumannii* adhesion obtained from the ratio between CFU/ml before and after infection. For statistical analyses, a non-parametric Mann–Whitney test was performed. ^∗∗^*p* = 0.0079, ns: not significant. **(D)** Representative immunofluorescence images obtained by confocal microscopy and analyzed with ImageJ of A549 cells infected with bacteria grown in solid. Images correspond to Z-projections at average intensity. *A. baumannii* strains were detected with an anti-*A. baumannii* antibody (green). The actin cytoskeleton and nucleus were labeled with phalloidin (white) and DAPI (blue), respectively. MOI, multiplicity of infection.

CdiA proteins can promote biofilm formation and/or attachment to eukaryotic cells (Rojas et al., 2002; Plamondon et al., 2007; Schmitt et al., 2007; Gottig et al., 2009; Neil and Apicella, 2009; Garcia et al., 2013; Ruhe et al., 2015; Mercy et al., 2016). To investigate whether *cdi*_1_ locus contributes to biofilm formation, we quantified in 96-well polystyrene plates over a 24-h time period the biofilm biomass formed by the wt and *cdiBAI*_1_ mutant strains. After 3 h, the capacity to form biofilm of the mutant was lower than that of the wt (Figure 7A). Surprisingly, *cdiBAI*_1_ mutant generated twice as much biofilm mass as the wt after 5 and 24 h (Figure 7A). Analysis of the depth bacterial growth on glass-bottom slides by confocal microscopy confirmed that the *cdiBAI*_1_ mutant exhibited the highest ability to form a bacterial 3D structured layer especially after 5 h (Figure 7B and Supplementary Figure S4). To determine whether the *cdi*_1_ locus is also implicated in the adhesion of *A. baumannii* DSM30011 to biotic surfaces, we compared the adhesiveness of wt and isogenic *cdiBAI*_1_ mutant strains to A549 epithelial cells by CFU measurement and confocal microscopy analysis to directly visualize *A. baumannii* strains and confirm CFU counts. No difference between strains was observed after 2 h of infection with exponential cultures grown in LB liquid medium (Figure 7C; exponential). The biofilm increase is a late phenotype (Figure 7A), and we noticed that on agar plate the Δ*cdiBAI*_1_ colonies were slightly smaller than those of the wt, reflecting a potential change in physiology depending on the growth condition. For these reasons, we directly analyzed the capacity of bacteria scratched from the agar plate to bind to host cells. As seen in Figure 7C (solid), the deletion of the *cdi*_1_ locus led to a significant 2.5-fold increase in the proportion of cell-associated bacteria 2-h post infection. Furthermore, the number of cell-attached bacteria detected by immunofluorescence using an anti-*Acinetobacter* antibody was also higher for the *cdiBAI*_1_ mutant (Figure 7D). Z-stack reconstruction confirmed that the bacteria are well attached to the cell surface and not endocyted by the host cells (Supplementary Figure S5).

## Discussion

The knowledge on virulence mechanisms and factors contributing to the pathogenic potential of *Acinetobacter baumannii* is limited, and a deeper understanding of its infection mechanisms may shed light on new strategies for drug development. On the basis of recent studies that characterized the potential implication of secretion systems including the TPS pathway, we performed a blast search using TpsA of diverse functions to evaluate their distribution within *A. baumannii*. The basic pattern reflected in this search was that several *cdi* loci could be identified in a large number of *A. baumannii* species and confirmed a previous study (De Gregorio et al., 2019). Strikingly, other subfamilies of TPS systems were absent with the exception of HxuA homologues found in some *A. baumannii* strains, which are TpsA involved in iron acquisition (Cope et al., 1994, 1995, 1998). These findings suggest that CDI systems may be significant players in *A. baumannii.* In this work, we used the environmental *A. baumannii* DSM30011 strain with two CDI systems.

Most of the *cdi* genes appear to be under tight regulatory control (Mercy et al., 2016) or only expressed during infection (Rojas et al., 2002; Aoki et al., 2010). Our results show that *A. baumannii* DSM30011 cells differentially produce its CDI systems. Indeed, *cdi*_2_ locus is not expressed under the rich medium and growth conditions used in this study and undergoes negative regulation. Interestingly, we noticed the presence of a putative pho box in the *cdiB*_2_ promoter region, suggesting a potential regulation by the transcriptional regulator PhoB through a differential phosphate level, as it has already been characterized for the *Pseudomonas aeruginosa tps* genes (Faure et al., 2013). Although additional experiments will be necessary to identify the regulatory circuits controlling the expression of this *cdiA* gene and its role in *A. baumannii*, we were able to show that CdiA_2_-CT, produced intracellularly in *Escherichia coli*, induces multiple DNA damages that the target cell cannot repair, leading to its death. This finding is consistent with the presence of a Tox-REase-7-fold domain and strongly suggests that CdiA_2_-CT functions as a cytoplasmic DNase to degrade nucleic acids like several other CdiA toxins (Willett et al., 2015). In contrast, the CdiA_1_ protein is highly secreted in *A. baumannii*, and the expression of *cdiBAI*_1_ genes is quite homogeneous within the population, in comparison with *Burkholderia thailandensis*, which expresses a high level of *cdi* genes in a stochastic manner (Anderson et al., 2012). In addition, in the *A. baumannii* SDF strain, we also observed the presence of two CDI systems, one of which is not repressed (Supplementary Figures S6, S7), and other studies have shown the constitutive activation of CDI systems within several *Acinetobacter* species in growth laboratory conditions (Perez et al., 2007; Darvish Alipour Astaneh et al., 2014; Harding et al., 2017), suggesting that *Acinetobacter* might not keep CDI system in an inactive state. Interestingly, we and others have shown that several *Acinetobacter* species often coproduce the CDI and the T6SS (Carruthers et al., 2013; Repizo et al., 2015; Harding et al., 2017; unpublished data), and in *Pseudomonas aeruginosa*, they are both regulated by the post-transcriptional RsmA regulator (Mercy et al., 2016; Allsopp et al., 2017). These observations might reflect that a co-regulation exists between these two systems, but additional work is needed to understand the real link between CDI and T6SS co-regulation in *Acinetobacter*.

Although we established that the CDI_1_ system is functional and arrests the growth of neighboring *A. baumannii* bacteria that do not contain the *cdi*_1_ locus, we have not yet investigated the mechanisms involved. However, we have shown that the production of CdiA_1_-CT in the cytoplasm of *E. coli* stops bacterial growth by inhibiting cell division rather than cell death, indicating that this domain might be responsible for the *A. baumannii* growth arrest during bacterial competition. CdiA_1_-CT does not contain any known conserved domains, but the use of fluorescent proteins reporting chromosome compaction state and DNA damage allowed us to exclude that this toxin functions as a DNase. The induction of growth arrest by CdiA_1_-CT can arise from different mechanisms of action. Many CdiA toxins act as nucleases that degrade tRNA or rRNA arresting growth by blocking the translation (Willett et al., 2015), whereas CdiA^EC93^ from *E. coli* EC93, a ionophore that dissipates the proton motive force, inhibits the growth by depleting ATP levels (Aoki et al., 2009). Further investigation will be necessary to identify the functional activity of CdiA_1_ toxin of *A. baumannii* DSM30011.

CdiA proteins might be multifunctional, which would be quite conceivable given their large sizes and might therefore have a broader role than bacterial competition. Indeed, studies in *A. baumannii* but also in other bacteria show that CdiA functions as adhesins mediating adhesion to epithelial cells or structuring a biofilm through bacteria–bacteria interactions (Balder et al., 2007; Plamondon et al., 2007; Darvish Alipour Astaneh et al., 2014; Ruhe et al., 2015; Pérez et al., 2016). Interestingly, CdiA_1_ of *A. baumannii* DSM30011 is a very large protein whose amino acid sequence is mostly constituted of β-helical and FHA repeats found in a number of TpsA adhesins (Jacob-Dubuisson et al., 2001). However, we found that CDI_1_ system does not promote biofilm formation nor adhesion to eukaryotic cells, but instead, its absence increases the adhesiveness of *A. baumannii*. The mechanism enabling the limitation of cell–cell or surface adhesion by the CDI_1_ system is not yet understood and remains to be discovered. Recent studies suggest a role for CDI beyond bacterial competition in collective behavior between sibling immune cells. In *P. aeruginosa*, the deletion of *cdi* locus increases the production of cyanide and swarming motility potentially via post-transcriptional regulation (Melvin et al., 2017). *Burkholderia* uses the contact-dependent signaling (CDS) mechanism to modulate gene expression in immune recipient bacteria that are dependent on the activity of the CdiA-CT toxin but independent of CDI growth inhibitory function (Garcia et al., 2016); and in *E. coli*, CDI modulates the cellular (p)ppGpp levels to increase the number of persister cells (Ghosh et al., 2018). It is therefore possible that the *A. baumannii* CDI_1_ system could also impact overall bacterial physiology by fine-tuning cellular responses.

## Experimental Procedures

### Growth Conditions, Strains, Plasmids, and Primers

Strains/plasmids and primers used in this study are listed in Supplementary Tables S1, S2, respectively. *Acinetobacter baumannii* and *Escherichia coli* strains were grown in LB, EZ-Rich Defined Medium (RDM), or M9 medium containing 0.2% glucose and 0.4% casamino acid (M9-casa) supplemented with appropriate antibiotics: 30 μg/ml of chloramphenicol (Cm), 50 μg/ml of ampicillin (Amp), 50 μg/ml of kanamycin (Kn), 15 μg/ml of gentamycin (Gm), and 50 (liquid) or 200 μg/ml (solid) of carbenicillin (Cb).

### Strain Construction

Gene insertion in the *E. coli* chromosome was performed by λRed recombination (Datsenko and Wanner, 2000; Yu et al., 2000). Mutant alleles were transferred by phage P1 transduction to generate the final strains. The *kan* and *cat* genes were removed using site-specific recombination induced by expression of the Flp recombinase from plasmid pCP20 (Datsenko and Wanner, 2000).

*cdiBAI*_1_ mutants were constructed following the Tucker et al. (2014) protocol. Briefly, the FRT (Flippase Recognition Target) site-flanked kanamycin resistance cassette was amplified from the pKD4 plasmid with primers containing 100-nt extension with homology to the flanking regions of *cdiBAI* locus. After the PCR product was electroporated into *A. baumannii* competent cells carrying pAT02 plasmid, which produces the RecA_b_ recombinase, mutants were selected on Kn 50 μg/ml, and the presence of integrated kanamycin cassette was verified by PCR. The *kan* gene was removed using site-specific recombination induced by expression of the Flp recombinase from plasmid pFLP2 (Hoang et al., 2000).

*cdiA*_1_ and *cdiB*_1_ mutants were constructed by amplifying 2 kb with homology to the flanking regions of the genes. PCR products were combined by overlapping extension PCR and cloned into pUC18T-mini-Tn7-Ap *Sac*I/*Bam*HI. The apramycin (Apr) resistance cassette was then amplified from pMHL2-2 and cloned between the 2-kb-flanking regions with *Nco*I/*Sac*I restriction site. Then, the 2-kb-flanked apramycin cassette was amplified by PCR and electroporated into *A. baumannii* competent cells, and mutants were selected on Apr 50 μg/ml. The presence of integrated cassette was verified by PCR.

### Toxicity Assays in *E. coli*

CdiA-CT domains were cloned with the artificial Shine-Dalgarno sequence into the pBAD33 plasmid using *Sac*I and *Sal*I. The *cdiI* genes were PCR amplified with a reverse primer encoding a 6xHis C-terminal tag and cloned into the pTrc99a plasmid using *Nco*I and *Bam*HI. Plasmid cloning was verified by Sanger sequencing (GATC Biotech). To perform toxicity assay, an overnight culture in LB with 0.5% glucose was washed in LB and diluted to an OD_600_ ∼ 0.05 in LB with 100 μM of IPTG and 1% arabinose to produce the immunity protein and the CdiA-CT domain, respectively. At indicated time of culture at 37°C, cells were washed in LB, and CFU/ml was calculated by plating onto LB agar plates containing Cm, Amp, and glucose 0.5%. The production of immunity proteins was verified by Western blot analyses. Briefly, the cell extract was separated on 12% SDS-PAGE, transferred onto polyvinylidene difluoride (PVDF) membranes, and probed with primary mouse anti-pentaHis (Qiagen) or anti EF-Tu.

### Live-Cell Microscopy Experiment

#### Live and Dead Assay

Overnight cultures in RDM supplemented with 0.5% glucose were washed and diluted to OD_600_ of 0.05 with 100 μM of IPTG and 1% arabinose and grown further at 37°C for 3 h 30 min before treatment with the LIVE/DEAD^®^ BacLight^TM^ Bacterial Viability Kit. To control the functionality of the Live/Dead assay, we treated the samples with ethanol to kill the bacteria, and as expected, the percentage of cell death reaches ∼100% in this condition (Supplementary Figure S2B). Cells were stained for 20 min, washed, resuspended in RDM, and spread over a RDM or M9-casa 1% agarose pad.

#### Time-Lapse Experiments

Overnight cultures in RDM or M9-casa media supplemented with 0.5% glucose were diluted to OD_600_ of 0.05 with 0.5% glucose and grown further to OD_600_ = 0.1. Cultures were loaded to B04A microfluidic chamber (ONIX, CellASIC^®^) with 5 psi for 1 min. Medium supplemented with 1% arabinose was maintained at 1 psi with a constant temperature of 37°C. Cells were imaged every 10 min for 6 h.

#### Image Acquisition and Analysis

Conventional wide-field fluorescence microscopy imaging was carried out on an Eclipse Ti-E microscope (Nikon), equipped with ×100/1.45 oil Plan Apo Lambda phase objective, FLash4 V2 CMOS camera (Hamamatsu), and using NIS software for image acquisition. Acquisition setting was 10 ms for Syto9, 100 ms for GFP, 10 ms for propidium iodide, and 100 ms for mCherry, using 50% power of a fluo LED Spectra X light source at 488- and 560-nm excitation wavelengths, respectively. Cell counting and length analysis were performed using MicrobeJ (Ducret et al., 2016).

### Quantification of Promoter Activities

The P_empty_ transcriptional fusion was constructed by cloning the *gfpmut2* gene from pUA66 plasmid as an *Eco*RI/*Bgl*II PCR fragment cloned into pWH1266. To construct transcriptional fusions, 500 bp corresponding to the putative promoter regions was amplified as a *Bam*HI/*Sac*I PCR fragment and cloned into pWH1266-P_empty_-*gfp*. To quantify promoter activities, overnight cultures in LB were diluted to an OD_600_ of 0.05 and grown at 37°C for 6 h. Two hundred microliters of cells was transferred into well of a black 96-well plate (Greiner), and the absorbance at 600 nm and fluorescence (excitation 485 nm, emission 530 nm) were measured using TECAN Spark multimode plate reader. The relative fluorescence was expressed as the intensity of fluorescence divided by the absorbance at 600 nm after subtracting the values of blank sample. Fluorescence repartition of the P*cdiB_1_-gfp* was performed by diluting an overnight culture in RDM to an OD_600_ of 0.05 and grown further at 37°C to an OD_600_ ∼ 0.8 before spreading the cells over a RDM 1% agarose pad. The analysis was performed using MicrobeJ (Ducret et al., 2016).

### Detection of CdiA_1_

Cell extract and secreted fractions were prepared as follows. Bacterial cells from overnight culture grown in LB were harvested by centrifugation at 4,000 × *g* for 20 min, and pellets (cell extracts) were resuspended in loading buffer. The supernatant fraction was centrifuged at 13,000 × *g* for 20 min at 4°C. Proteins from the supernatant (secreted fraction) were then precipitated with 12% (w/v) trichloroacetic acid (TCA), washed with acetone, air-dried, and resuspended in loading buffer. The protein samples were then heated to 95°C for 10 min, separated by SDS-PAGE, and revealed by Coomassie blue staining or immunoblotting.

### Growth Competition Assays

The attackers and target strains were grown overnight separately in LB medium without antibiotic at 37°C. Overnight cultures were diluted to an OD_600_ of 0.05 in LB medium without antibiotic and grown at 37°C with shaking to an OD_600_ ∼ 0.35. Strains were then mixed at an attacker to target cell ratio of 4:1, and the mixed bacteria were grown at 37°C with agitation at 160 rpm. At indicated times, the mix was serially diluted and plated onto kanamycin-containing LB agar plates to determine the CFU/ml of the target strain.

### Biofilm Assays

Biofilm assays were performed following the O’Toole (2011) protocol. Briefly, overnight cultures were inoculated into LB at a final OD_600_ of 0.1. One hundred microliters of culture was added to 96-well polystyrene plates and incubated over a 5-h period without shaking. At the indicated times, cells in suspension were removed, and the wells washed twice with distilled water. One hundred twenty-five microliters of 0.1% crystal violet was then added to the wells, incubated for 10 min, and washed three times with 125 μl of distilled water. To solubilize the crystal violet, 125 μl of 30% acetic acid was added and the absorbance measured at 550 nm. The ratio OD_550_/OD_600_ was calculated to normalize the biofilm formation due to variation in bacterial growth.

For biofilm imaging, bacteria were grown in BM2 minimal medium (Repizo et al., 2015) supplemented with 10 mM of potassium glutamate (BM2G) as carbon source in a glass-bottom 24-well μ-plate. Cultures were inoculated at an initial OD_600_ of 0.1 from an overnight culture grown in LB and then incubated at 37°C under static conditions. At indicated times, the biofilm was labeled with DAPI, and images were taken with a confocal Zeiss LSM800 Airyscan microscope and analyzed with ImageJ and Imaris software.

#### *A. baumannii* Infection of A549 Cells

A549 cells were grown in Dulbecco’s modified Eagle medium (DMEM) supplemented with 10% of fetal calf serum and 1% L-glutamine at 37°C with 5% CO_2_ atmosphere. For adhesion assays and microscopy analysis, A549 cells were first seeded into 24-well tissue culture plates at 5 × 10^5^ cells/well to obtain a monolayer. *A. baumannii* strains were grown on LB agar plate for 24 h at 37°C to form a “lawn” covering. Bacteria were scraped from the agar surface and resuspended in 1 ml of LB to form a homogeneous suspension of ∼10^8^ CFU/ml. Cells were then infected at a multiplicity of infection (MOI) of 100 of *A. baumannii* in 500 μl of complete medium per well. Plates were centrifuged at 400 × *g* for 5 min and then incubated for 2 h at 37°C with 5% CO_2_ atmosphere. Cells were then washed five times with phosphate-buffered saline (PBS) 1 × and either lysed with 0.1% sodium deoxycholate (5-min incubation) or fixed with 3% paraformaldehyde (15 min incubation). The number of CFU/ml before and after infection was determined to calculate the percentage of bacterial adhesion. The number of CFU/ml of the inocula was also used to verify the MOI and ensure equivalent numbers of bacteria were used for wt and mutant strains.

### Immunofluorescence Labeling and A549 Cell Microscopy

After fixation, cells were incubated at room temperature for 1 h in a PBS 1 × 0.1% saponin and 10% horse serum solution for permeabilization and blocking. Cells were then labeled at room temperature with primary rabbit anti-*Acinetobacter* antibody mix diluted at 1/20,000 in the same solution for 1 h followed by two washes in PBS 1 × 0.1% saponin. Secondary antibodies (anti-rabbit Alexa-488 [1/1,000], phalloidin-568 [1/250], and DAPI nuclear dye [1/1,000] to label *A. baumannii*, actin cytoskeleton, and host cell nucleus, respectively) were then mixed and incubated for 45 min, followed by two washes in PBS 1 × 0.1% saponin, one wash in PBS 1×, and one wash in distilled water before mounting with ProLong Gold. Coverslips were examined on a Zeiss LSM800 laser scanning confocal microscopes and analyzed with ImageJ software (Schindelin et al., 2012). Z-stack tool and Z-projection for maximum intensity were used for image presentation.

## Data Availability Statement

All datasets generated for this study are included in the article/Supplementary Material.

## Author Contributions

MR performed the majority of the experiments. SR, LC, JC, and CL also conducted the experiments. MR, SS, and CL participated in the experimental design and data analysis. SB conceived the project, designed and undertook experiments, interpreted data, and wrote the manuscript. All authors read and approved the manuscript.

## Conflict of Interest

The authors declare that the research was conducted in the absence of any commercial or financial relationships that could be construed as a potential conflict of interest.
