# Supplementary material for: Identification of a Contact-Dependent Growth Inhibition (CDI) System That Reduces Biofilm Formation and Host Cell Adhesion of Acinetobacter baumannii DSM30011 Strain
Source: Front Microbiol. 2019 Oct 30;10:2450. doi: 10.3389/fmicb.2019.02450 (PMC6831553; doi:10.3389/fmicb.2019.02450)
Supplement: Supplementary file 9 [file Data_Sheet_1.pdf]

Identification of a CDI system that reduces biofilm formation and host cell adhesion of *Acinetobacter baumannii* DSM30011 strain

Morgane Roussin[1], Sedera Rabarioelina[1], Laurence Cluzeau[1], Julien Cayron[2], Christian Lesterlin[2], Suzana P. Salcedo[1*] and Sarah Bigot[1,2*]

Supplementary data

Supplementary Figure legends

Supplementary Figure S1. Effect of $CdiA_2$-CT production on *E. coli* cell viability and filamentation. (A) The cell viability of *E. coli* DH5α strain producing $CdiA_2$-CT with or without $CdiI_2$ was estimated in colony forming unit (CFU) /ml. Means from three biological replicates are plotted. (B) Cell length repartition of *E. coli* DH5α (*recA*$^-$) and RecA-GFP producing MG1655 (*recA*$^+$) 3 h after induction of $CdiA_2$-CT toxin. n indicate the number of analysed cells.

Supplementary Figure S2. The $CdiA_1$-CT producing *E. coli* strain are viable cells. (A) HU-mCherry producing *E. coli* MG1655 growth monitored by measuring the optical density at 600 nm (dotted lines) and colony forming unit (CFU) /ml (bars) after induction of $CdiA_1$-CT in presence or in absence of $CdiI_1$. Means from three biological replicates are plotted. (B) Live and dead assay performed with RecA-GFP *E. coli* MG1655 strain 3 h after induction of $CdiA_1$-CT in presence or absence of $CdiI_1$. Before staining, the bacteria were treated with or without (w/o) ethanol. Left panel: representative fluorescence microscopy of $CdiA_1$-CT producing cells

after staining with SYTO9 and Propidium iodide (PI). Scale bar corresponds to 1 μm; right panel: percentage of dead cells. n indicate the number of analysed cells.

Supplementary Figure S3. Growth analysis of *A. baumannii* DSM30011 wild-type and mutant strains. The growth was monitored in LB medium by measurement of optical density at 600 nm (OD_{600}) (solid lines) and colony forming unit (CFU) /ml (bars).

Supplementary Figure S4. Confocal microscopy images correspond to 3D projections of the maximum intensities obtained from z-stacks of biofilms formed by the wild-type and Δ$cdiBAI_1$ strains on glass-bottom slides at 3, 5 and 24h. Bacteria were labelled with DAPI. Scale bar 10 μm.

Supplementary Figure S5. Z-stack analysis of *A. baumannii* adhesion to lung epithelial cells. Confocal microscopy of A549 cells infected with *A. baumannii* DSM30011 wild-type and Δ$cdiBAI_1$ strains during 2 hours. The corresponding axis are shown. Bacteria were labeled with an anti-*Acinetobacter* antibody (green), the actin cytoskeleton with phalloidin (grey) and nuclei with DAPI (blue).

Supplementary Figure S6. The $CdiA_1^{AbSDF}$ is produced in *A. baumannii* SDF strain. (A) Schematic of the cdi_1^{AbSDF} and cdi_2^{AbSDF} loci. Genes encoding putative CdiB transporters, CdiA exoproteins and CdiI immunity proteins are colored respectively in brown, green and orange. *cdiC* genes shown in red encode proteins that share high homologies with HlyC acyltransferases that activate the α-hemolysin HlyA in *E. coli* through fatty acylation using acyl-acyl carrier protein (acyl-ACP) as the fatty acid donor (Ogier et al., 2016). cdi_1^{AbSDF} locus refers to *ABSDF_RS16855*, *ABSDF_RS16850*, *ABSDF_RS16845, ABSDF_RS16840* genes and cdi_2^{AbSDF} to *ABSDF_RS16500, ABSDF_RS16505, ABSDF_RS16510*, ABSDF_RS16515*. * indicates the truncated version of the annotated gene on the NCBI database; the 3'-end of the gene was sequenced and the result is shown in Supplementary Figure S7. (B) Cell extract (CE) of wild-type strain grown overnight was analyzed by blue Coomassie staining. Arrow indicates

the $CdiA_1^{AbSDF}$ protein identified by mass spectrometry. Molecular weight marker (kDa) is indicated on the left.

Supplementary Figure S7. Sequence of complete *ABSDF_RS16510* gene in *A. baumannii* SDF strain. Bold sequence shows the annotated gene on the NCBI database, italic the intergenic sequence and the start codon of the potential *$cdiI_2^{AbSDF}$* (*ABSDF_RS16515*) immunity gene is underlined.

Movie 1. Time-lapse fluorescence microscopy of the recombinant HU-mCherry protein produced by *E. coli* MG1655 strain after induction of the $CdiA_2$-CT.

Movie 2. Time-lapse fluorescence microscopy of the recombinant HU-mCherry protein produced by *E. coli* MG1655 strain after induction of the $CdiA_2$-CT in presence of its cognate $CdiI_2$ immunity protein.

Movie 3. Time-lapse fluorescence microscopy of the recombinant RecA-GFP protein produced by *E. coli* MG1655 strain after induction of the $CdiA_2$-CT.

Movie 4. Time-lapse fluorescence microscopy of the recombinant RecA-GFP protein produced by *E. coli* MG1655 strain after induction of the $CdiA_2$-CT in presence of its cognate $CdiI_2$ immunity protein.

Movie 5. Time-lapse fluorescence microscopy of the recombinant HU-mCherry protein produced by *E. coli* MG1655 strain after induction of the $CdiA_1$-CT.

Movie 6. Time-lapse fluorescence microscopy of the recombinant RecA-GFP protein produced by *E. coli* MG1655 strain after induction of the $CdiA_1$-CT.

Movie 7. Time-lapse fluorescence microscopy of the recombinant HU-mCherry protein produced by *E. coli* MG1655 strain after induction of the $CdiA_1$-CT in presence of its cognate $CdiI_1$ immunity protein.

Movie 8. Time-lapse fluorescence microscopy of the recombinant RecA-GFP protein produced by *E. coli* MG1655 strain after induction of the $CdiA_1$-CT in presence of its cognate $CdiI_1$ immunity protein.

Supplementary Table S1. Plasmids and strains used in this study

Plasmids	Description	Source
pKD3	Carries *FRT-Cm-FRT* used for λred integration ; Cm^R	(Datsenko and Wanner, 2000)
pKD3-AS-*recA-Cm*	Carries *recA* gene under its own promoter used for LY653 construction; Cm^R	This study
pKD4	Carries *FRT-kan-FRT* used for RecET integration; Kn^R	(Datsenko and Wanner, 2000)
pCP20	Site-specific excision vector, Flp recombinase expression; Ap^R	(Datsenko and Wanner, 2000)
pAT02	Expresses the Rec_{Ab} recombinase; Ap^R	(Tucker et al., 2014)
pFLP2	Site-specific excision vector, Flp recombinase expression; Ap^R	(Hoang et al., 2000)
pMHL2-2	Apramycin cassette, $Apra^R$	(Godeux et al., 2018)
pUA66	*gfpmut2* gene under the control of *rrnB* promoter; Kn^R	(Zaslaver et al., 2006)
pWH1266	Expression plasmid, *E. coli-Acinetobacter* shuttle plasmid; Ap^R	(Hunger et al., 1990)
pWH1266-P_{empty}-*gfp*	pWH1266 containing promoterless *gfp* gene; Ap^R	This study
pWH1266-P*cdiB$_1$-gfp*	pWH1266 containing 500 bp upstream *cdiB$_1$* gene from *A. baumannii* DSM30011 fused to *gfp* gene; Ap^R	This study
pWH1266-P*cdiA$_1$-gfp*	pWH1266 containing 500 bp upstream *cdiA$_1$* gene from *A. baumannii* DSM30011 fused to *gfp* gene; Ap^R	This study
pWH1266-P*cdiI$_1$-gfp*	pWH1266 containing 500 bp upstream *cdiI$_1$* gene from *A. baumannii* DSM30011 fused to *gfp* gene; Ap^R	This study
pWH1266-P*cdiB$_2$-gfp*	pWH1266 containing 500 bp upstream *cdiB$_2$* gene from *A. baumannii* DSM30011 fused to *gfp* gene; Ap^R	This study
pWH1266-P*cdiA$_2$-gfp*	pWH1266 containing 500 bp upstream *cdiA$_2$* gene from *A. baumannii* DSM30011 fused to *gfp* gene; Ap^R	This study
pWH1266-P*cdiI$_2$-gfp*	pWH1266 containing 500 bp upstream *cdiI$_2$* gene from *A. baumannii* DSM30011 fused to *gfp* gene; Ap^R	This study
pUC18T-mini-Tn7T-Gm	mini-Tn*7*T based vector; mobilizable; Ap^R Gm^R	(Choi and Schweizer, 2006)
pUC18T-mini-Tn7-Ap	Gm^R removed from pUC18T-mini-Tn7T-Gm by *Xba*I digestion and self-ligation; Ap^R	This study
pUC18T-Δ*cdiA$_1$*	2 kb with homology to the flanking regions of *cdiA$_1$*	This study
pUC18T-Δ*cdiA$_1$*-Apra	Apramycin cassette flanked by 2kb upstream and downstream of *cdiA$_1$*; Ap^R, $Apra^R$	This study
pUC18T-Δ*cdiB$_1$*	2 kb with homology to the flanking regions of *cdiB$_1$*	This study
pUC18T-Δ*cdiB$_1$*-Apra	Apramycin cassette flanked by 2kb upstream and downstream of *cdiB$_1$*; Ap^R, $Apra^R$	This study
pBAD33	Arabinose inducible expression plasmid, Cm^R	(Guzman et al., 1995)
pBAD33-$CdiA_1$-CT	pBAD33 containing *cdiA-CT$_1^{Ab30011}$* toxin (residues Val^{4746}- Arg^{5104} of $CdiA_1^{Ab30011}$); Cm^R	This study
pBAD33-$CdiA_2$-CT	Expresses *cdiA-CT$_2^{Ab30011}$* toxin (residues Ala^{1715}- Tyr^{1950} of $CdiA_2^{Ab30011}$); Cm^R	This study
pTrc99a	IPTG-inducible expression plasmid; Ap^R	GE healthcare
pTrc99a-$CdiI_1$-His_6	IPTG-inducible expression of $CdiI_1^{Ab30011}$-His_6 immunity gene; Ap^R	This study

pTrc99a-CdiI$_2$-His$_6$	IPTG-inducible expression of CdiI$_2^{Ab30011}$-His$_6$ immunity gene; Ap^R	This study
**Strains**	**Description**	**Source**
E. coli		
Top10	General cloning<br>*F- mcrA Δ(mrr-hsdRMS-mcrBC) φ80lacZΔM15 ΔlacX74 nupG recA1 araD139 Δ(ara-leu)7697 galE15 galK16 rpsL(Str^R) endA1 λ$^-$*	Invitrogen
DH5α	General cloning and toxicity assay<br>F$^-$ *endA1 glnV44 thi-1 recA1 relA1 gyrA96 deoR nupG purB20* φ80d*lacZ*ΔM15 Δ(*lacZYA-argF*)U169, hsdR17($r_K^- m_K^+$), λ	Lab collection
BW25141	*Δ(araD-araB)567*, *ΔlacZ4787*(::rrnB-3), *Δ(phoB-phoR)580*, *λ$^-$*, *galU95*, *ΔuidA3::pir$^+$*, *recA1*, *endA9*(del-ins)*::FRT*, *rph-1*, *Δ(rhaD-rhaB)568*, *hsdR514*	Lab collection
DY330	W3110 Δ*lacU169, gal490, λcI857,* Δ*(cro-bioA)*	(Yu et al., 2000)
MG1655	λ$^-$, *rph-1*	Coli Genetic Stock Center (CGSC) #6300
MS388	MG1655 *rpsL$^+$*, Str^R	Gift from F. Cornet
OX468	W1485 F- *leu thyA thi deoB* or *C supE rpsL*, *hupA-mcherry-FRT-kn-FRT,* Str^R, Kn^R	Gift from F. Cornet
LY119	MS388 *hupA-mcherry-FRT-kn-FRT*, Str^R, Kn^R	This study; MS388 x P1.OX468
LY248	MS388 *hupA-mcherry*, Str^R	This study; derivative of LY119, *kn* removed via pCP20
LY653	DY330 *fhuB::recA-FRT-Cm-FRT*	This study; λred *recA* at the *fhuB* chromosomal locus
SS3085	*recA4155-gfp-Kn(4155,4136),* Kn^R	(Renzette et al., 2005)
MS388 *recA4155-gfp-Kn*	MS388 *recA4155-gfp-Kn(4155,4136),* Kn^R	This study; MS388 x P1.SS3085
LY769	MS388 *recA4155-gfp-Kn, fhuB::recA-FRT-Cm-FRT,* Str^R, Kn^R, Cm^R	This study; MS388 *recA4155-gfp-Kn* x P1.LY653
LY844	MS388 *recA4155-gfp-Kn, fhuB::recA-FRT,* Str^R, Kn^R	This study; derivative of LY769, *Cm* removed via pCP20
A. baumannii		
DSM30011	Wild-type	(Repizo et al., 2017)
Δ*cdiA$_1$*	DSM30011 Δ*cdiA$_1$::apra,* Apra^R	This study
Δ*cdiB$_1$*	DSM30011 Δ*cdiB$_1$::apra,* Apra^R	This study
Δ*cdiBAI$_1$::kn*	DSM30011 Δ*cdiA$_1$::FRT-kn-FRT,* Kn^R	This study
Δ*cdiBAI$_1$*	DSM30011 Δ*cdiA$_1$::FRT*	This study; derivative of Δ*cdiBAI$_1$::kn*, *kn* removed by pFLP2

Ap^R, Cm^R, Kn^R, Str^R, Apra^R, Gm^R resistance to ampicillin, chloramphenicol, kanamycin, streptomycin, apramycin and gentamycin respectively.

Supplementary Table S2. Primers used for plasmid and strain constructions

Name	sequence	construct
Ol359	GGAATAGGAACTAAGGAGGAGAATGCTTCAGCGGCGAC	pKD3-AS-*recA-Cm* construct using Gibson Assembly; PCR on MS388 (Ol359/Ol360) and pKD3 (Ol361/Ol362)
Ol360	AGCCATGGTCCATATGAATACGCAGATGCGACCCTTGTG	
Ol361	TCCTCCTTAGTTCCTATTCC	
Ol362	TATTCATATGGACCATGGCT	
P363	CGCCAGCGTCGCATCGGGCATCCGTTGTCGCGTAAACGCTCTTGAGCGATTGTGTAGGC	LY653 strain construct using λred; PCR on pKD3-recA-FRT-Cm-FRT
P364	TTCCATATAACGGCCCTGTACGCCTGGACGGATAAAACGCAGATGCGACCCTTGTG	
Big3	GGGCTAGCGAATTCGAGCTCAGGAGGAATTCACCATGGTTGAGAACAATTTATTTGG	pBAD33-CdiA$_1$-CT plasmid construct using SLIC; PCR on DSM30011
Big4	TTGCATGCCTGCAGGTCGACTTAACGACGTTTTAATGGG	
Big5	GGGCTAGCGAATTCGAGCTCAGGAGGAATTCACCATGGCAATCCAGAATAACTCTTT	pBAD33-CdiA$_2$-CT plasmid construct using SLIC; PCR on DSM30011.
Big6	TTGCATGCCTGCAGGTCGACCTAATATGGGTGGAGTGTCT	
Big7	ATTTCACACAGGAAACAGACCATGATAGATGTTAGCCCAGA	pTrc99a-CdiI$_1$-His$_6$ plasmid construct using SLIC; PCR on DSM30011
Big8	GGTCGACTCTAGAGGATCCTAGTGGTGGTGGTGGTGGTGAGCTTCGACCTTATATTTTG	
Big9	ATTTCACACAGGAAACAGACCATGAAGTTTAAACGCCCTAT	pTrc99a-CdiI$_2$-His$_6$ plasmid construct using SLIC. PCR on DSM30011
Big10	GGTCGACTCTAGAGGATCCTAGTGGTGGTGGTGGTGGTGCTTCGCTTTAAACGAGTTAA	
Big44	AATTCGATCATGCATGAGCTCCCGGGTGTCTAGCTGATTTTTATAA	pUC18T-Δ*cdiA$_1$* plasmid construct using SLIC. Overlapping PCR on DSM30011 (Big44/Big45 and Big46/Big47)
Big45	CCGCGGTAGTCCATTGACCATGGCATTCTAAAATCTCACTAAA	
Big46	CCATGGTCAATGGACTACCGCGGCGTCGTTAAGGAAAATAGGT	
Big47	TTCCTGCAGCCCGGGGGATCCTTGTATAGACCAATTATCAG	
Big84	TGAGATTTTAGAATGCCATGGAATCAAGGCCCGATCCTTGG	pUC18T-Δ*cdiA$_1$*-Apra plasmid construct using ligation
Big85	TTTTCCTTAACGACGCCGCGGCGCCAGGGTTTTCCCAGTCA	
Big88	AATTCGATCATGCATGAGCTCCCGGGCCCTCGAATTATGAATGCGA	pUC18T-Δ*cdiB$_1$* plasmid construct using SLIC; Overlapping PCR on DSM30011 (Big88/Big89 and Big90/Big91). Δ*cdiB$_1$* strain construct; PCR (Big88/Big91) on pUC18T-Δ*cdiB$_1$*-Apra plasmid
Big89	CCGCGGTAGTCCATTGACCATGGCATATAAAATTAAACCGTGA	
Big90	CCATGGTCAATGGACTACCGCGGTAATTCAAGTTTAAAGGTCC	
Big91	TTCCTGCAGCCCGGGGGATCCTGAATACACAACACCTGACT	
Big11	CGGACTATAGACTATACTAGTAAGTGCCTTTTAAAGTGGGT	Δ*cdiA$_1$* strain construct. PCR on pUC18T-Δ*cdiA$_1$*-Apra plasmid
Big14	CCCCTGCAGGTCGACGGATCCATAATTCAGGCATATGAG	
Big229	TTTTACATACAGACAAATCTGTATAAAAAATGCTTCCACTTTGTTAAGGTTTTGTTAAAATAAAATTAAGTTGATATTAATCACGGTTTAATTTTATatgTGTAGGCTGGAGCTGCTTCG	Δ*cdiBAI$_1$::kn* strain construct using Rec$_{Ab}$; PCR on pKD4
Big230	TAAATAATGAAAAGGTATCATTATTTCCCAAAATCATTATTTATAATTTTATTCCAGTTATTTATGTAAAAAAAGGTTAGAAATTCTTGAGTAACAATTACCTCCTTAGTTCCTATTCCG	

Supplementary Reference

Choi, K.-H., and Schweizer, H. P. (2006). mini-Tn7 insertion in bacteria with single attTn7 sites: example *Pseudomonas aeruginosa*. *Nat. Protoc.* 1, 153–161. doi:10.1038/nprot.2006.24.

Datsenko, K., and Wanner, B. (2000). One-step inactivation of chromosomal genes in *Escherichia coli* K-12 using PCR products. *Proc. Natl. Acad. Sci. U. S. A.* 97, 6640–6645.

Godeux, A.-S., Lupo, A., Haenni, M., Guette-Marquet, S., Wilharm, G., Laaberki, M.-H., et al. (2018). Fluorescence-Based Detection of Natural Transformation in Drug-Resistant *Acinetobacter baumannii*. *J. Bacteriol.* 200. doi:10.1128/JB.00181-18.

Guzman, L. M., Belin, D., Carson, M. J., and Beckwith, J. (1995). Tight regulation, modulation, and high-level expression by vectors containing the arabinose PBAD promoter. *J. Bacteriol.* 177, 4121–4130.

Hoang, T. T., Kutchma, A. J., Becher, A., and Schweizer, H. P. (2000). Integration-proficient plasmids for *Pseudomonas aeruginosa*: site-specific integration and use for engineering of reporter and expression strains. *Plasmid* 43, 59–72. doi:10.1006/plas.1999.1441.

Hunger, M., Schmucker, R., Kishan, V., and Hillen, W. (1990). Analysis and nucleotide sequence of an origin of DNA replication in *Acinetobacter calcoaceticus* and its use for Escherichia coli shuttle plasmids. *Gene* 87, 45–51.

Renzette, N., Gumlaw, N., Nordman, J. T., Krieger, M., Yeh, S.-P., Long, E., et al. (2005). Localization of RecA in *Escherichia coli* K-12 using RecA-GFP. *Mol. Microbiol.* 57, 1074–1085. doi:10.1111/j.1365-2958.2005.04755.x.

Repizo, G. D., Viale, A. M., Borges, V., Cameranesi, M. M., Taib, N., Espariz, M., et al. (2017). The Environmental *Acinetobacter baumannii* Isolate DSM30011 Reveals Clues into the Preantibiotic Era Genome Diversity, Virulence Potential, and Niche Range of a Predominant Nosocomial Pathogen. *Genome Biol. Evol.* 9, 2292–2307. doi:10.1093/gbe/evx162.

Tucker, A. T., Nowicki, E. M., Boll, J. M., Knauf, G. A., Burdis, N. C., Trent, M. S., et al. (2014). Defining Gene-Phenotype Relationships in *Acinetobacter baumannii* through One-Step Chromosomal Gene Inactivation. *mBio* 5, e01313-14-e01313-14. doi:10.1128/mBio.01313-14.

Yu, D., Ellis, H. M., Lee, E. C., Jenkins, N. A., Copeland, N. G., and Court, D. L. (2000). An efficient recombination system for chromosome engineering in *Escherichia coli*. *Proc. Natl. Acad. Sci. U. S. A.* 97, 5978–5983. doi:10.1073/pnas.100127597.

Zaslaver, A., Bren, A., Ronen, M., Itzkovitz, S., Kikoin, I., Shavit, S., et al. (2006). A comprehensive library of fluorescent transcriptional reporters for *Escherichia coli*. *Nat. Methods* 3, 623–628. doi:10.1038/nmeth895.

Supplementary Figure S1

A.

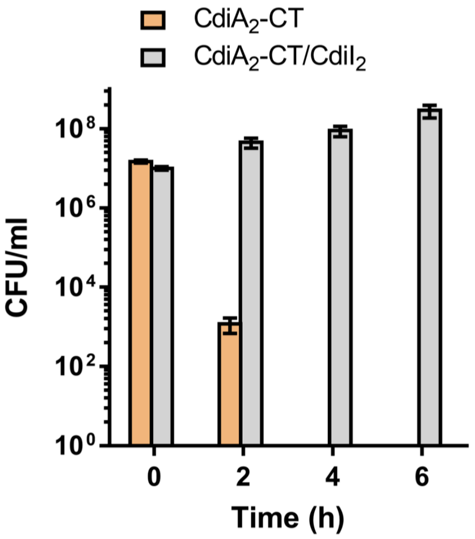

B.

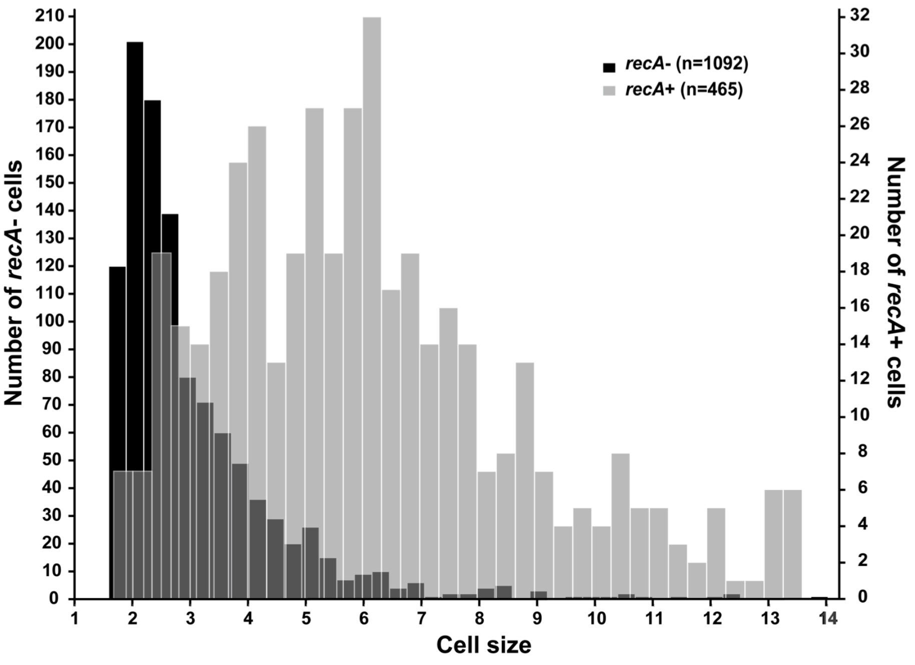

Supplementary Figure S2

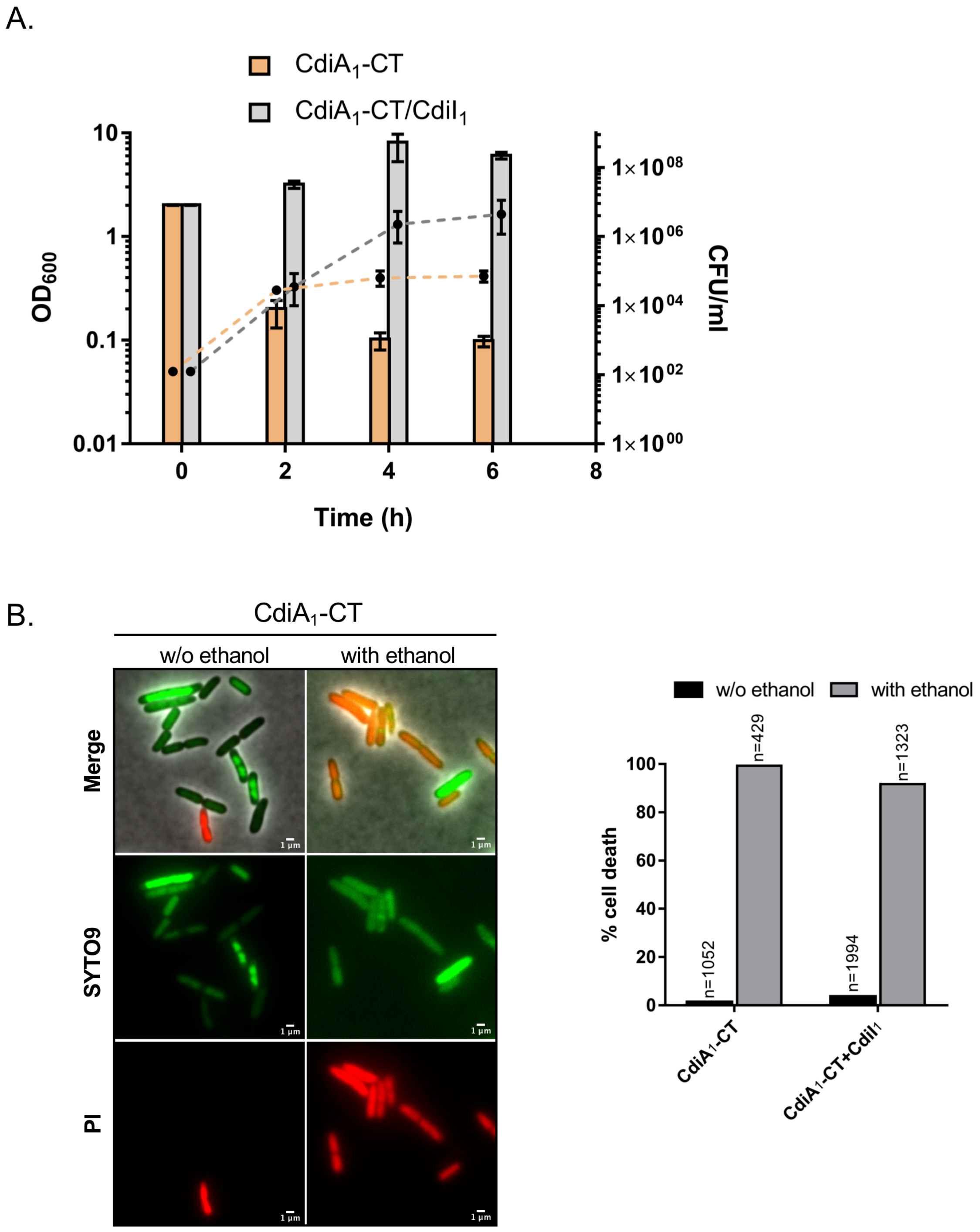

Supplementary Figure S3

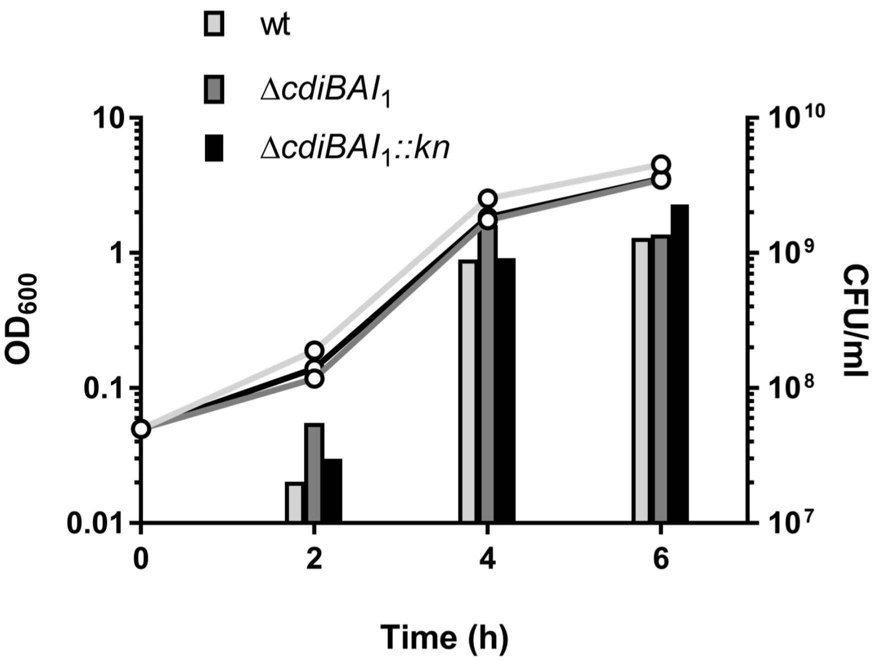

Supplementary Figure S4

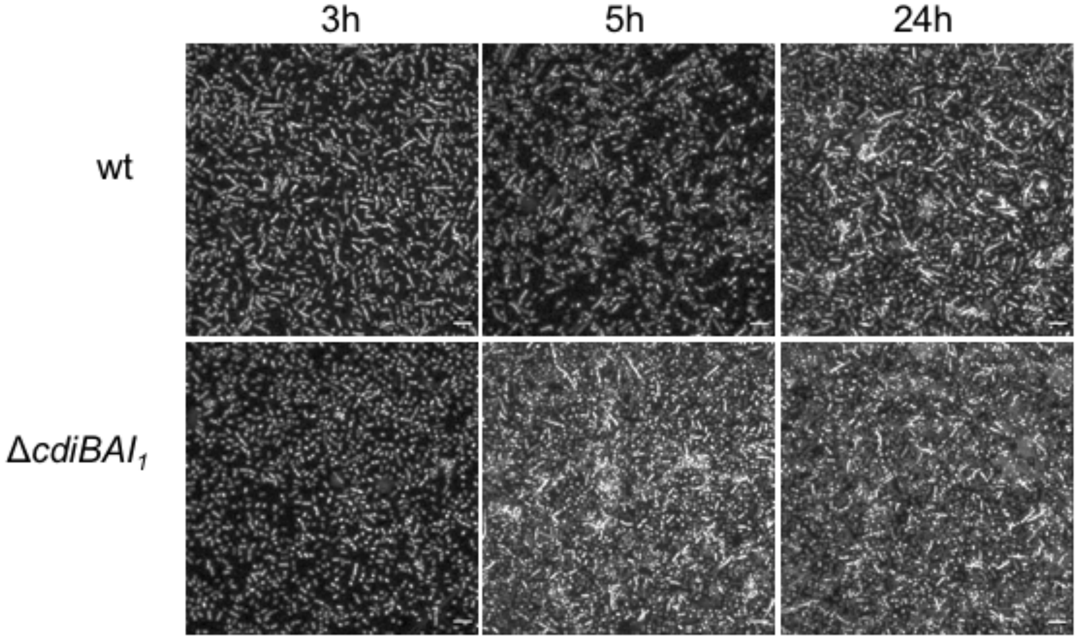

Supplementary Figure S5

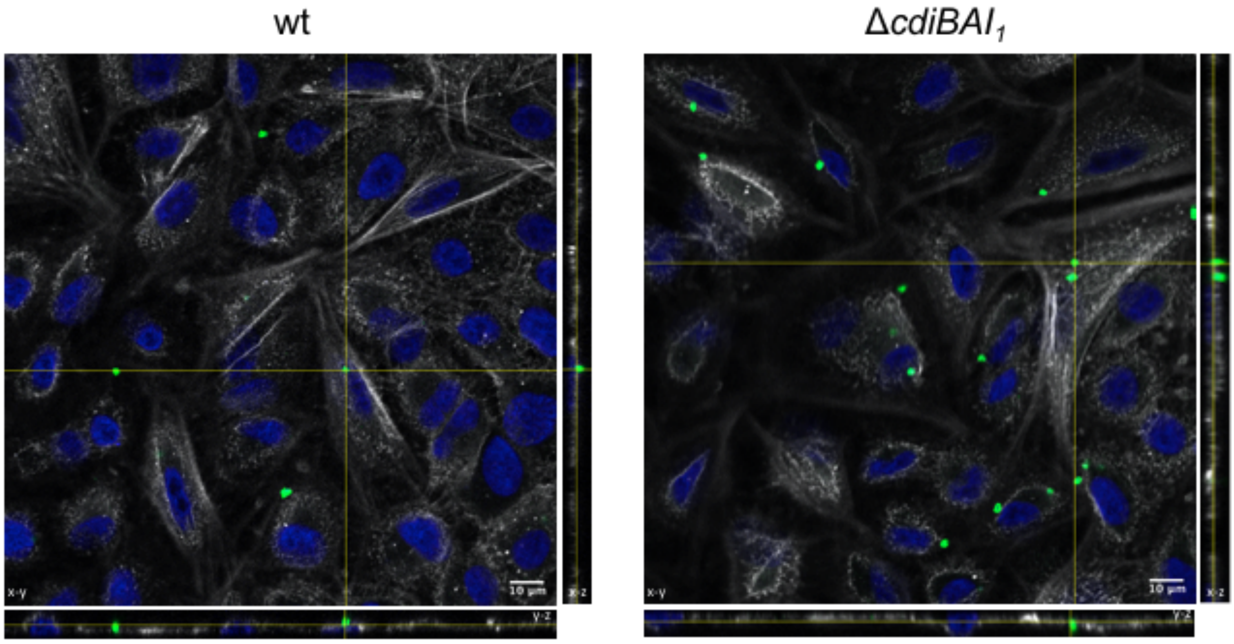

Supplementary Figure S6

A.

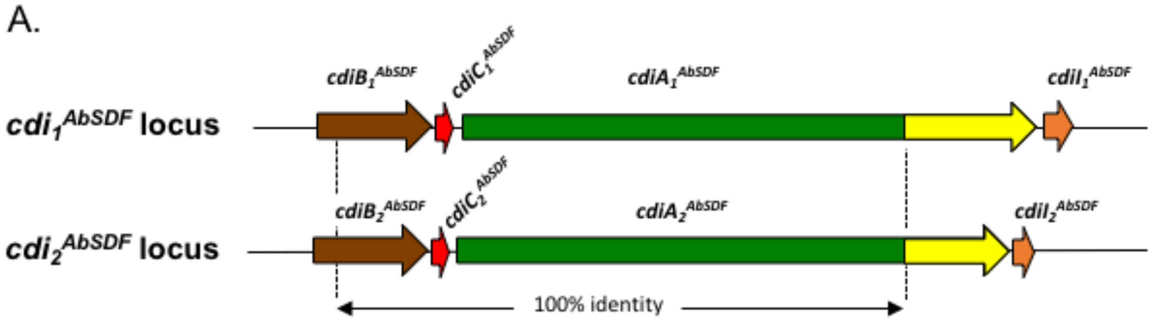

B.

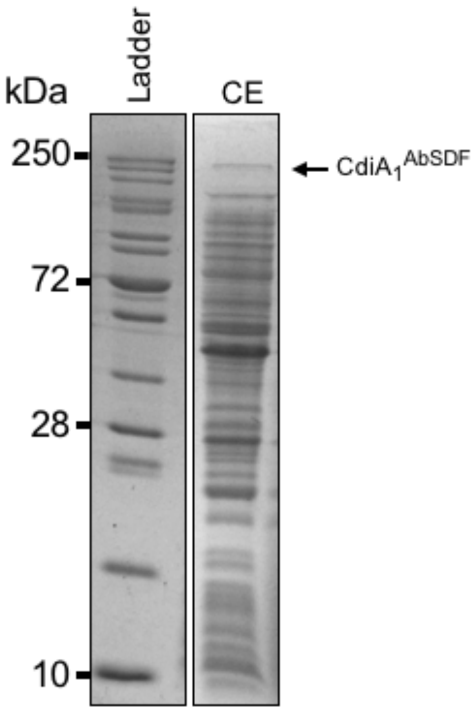

Supplementary Figure S7

cdiA$_{2\ AbSDF}$ (*ABSDF_RS16510)* gene region

ATGAATAAGAATCGTTATAGAGTGATATTTAGCCAAGCACGTGGCATGTTCATTGCGGTTGCAGAGATTGTAAAAAGTCGTACAAAAATGGCTG
GACAGAGCATAGCCGCAGGTAACGAGACTGTAACGAATTCACTTACACCGAACAGCTATAAAAAGTTGAATCCACTCCATTTTTCTGTGGTGAC
GCTATTAGGTGCAGTGGTGTATACCATTCCACTTTCTAGTATTGCAAATACCCAGATTCATGCTGACAAATCAGCACCTTCAAATCAACAAGCT
CAGATTCTCAACTCTGCCAATGGTACGGTTCAAGTCAATATTCAAACCCCAAGCGCTGGTGGCGTTTCTCGAAATACCTACAGCCAATTTGATG
TAGGGCAACAAGGTGCCATTCTAAACAACTCGCGCAATAACGTTCAGACCCAAATTGGTGGTTGGGTGCAGGGCAATGCAAACCTTGCAAGAGG
TGAAGCCAAAGTTATTCTCAATGAGGTCAACTCAAGTAATCCTAGCCAGCTTAGGGGCTATCTGGAAGTTGCAGGAAAATCTGCACAAGTAGTG
ATTGCCAATCCATCAGGTTTAGTCTGTGATGGCTGTGGGGTGATCAATGCAGACCGCTTTACTTTAACTACAGGTCAGGCGGTGATGAATCAAG
GTTATCTTGAGTCATTCCGAGTACGCGAAGGACAAGTCACCATTGAAGGCAAAGGTTTAAATGGGAGCCTGACGCCATATACCGATATCTATGC
GCGTGCATTAAAAGTGAATGCAGGTTTATACGCCAATGAGCTCAAAACAGTATTGGGTCAAAACGACATCAGTATTCAAGACCCATCTGCACCA
CAAATTGCCAAAGCAACGACAGGAACTAATACAACGCCTCAGCCAGACTTTGCCCTTGATGTGGGTCAATTGGGTGGCATGTACGCTGGAAAAA
TCTATTTAGTGGGAACTGAACAAGGCTTGGGTGTACGTAATGCGGGTTCAATGAACGCAACTACAGGTCAGTTGAGTTTGAATGCTAATGGAGA
CTTGATCAATACAGGTAATATGATTGCCAACAAAGACCAAATCGCCATTCAAGCGCAAAACATAAAAAACAGTGGAAATATCAGTAGTACACAA
CACAACATTCAAATCAAAGCCGATAACATTCAGAACAGTGGTTTGATTGCCACCAATGATGAAATCCAACTCAATACACAAGACAAGATTGATA
ATAATGACGGTGTCATCAATGCAGGACGGATTGACTTTACAGCGCAGACCCTCAGCAATGACAAAGGAAAAATTGAACAAACAGGCCAGCAACA
ACTCAATATCAGTGCAAAAACACTGGATAATACTCAAGGGTTGATTGGACAGGCGACGAAAGAGAGCAGTGGAACATCAGGCGGAACAACAACC
CCTAGCGTAACGGACCCGGAACAACAATCTTCAGCGCAAGACTCAAGTACCGTTGAAGTTGCACCAGTTGATCTGACCCCTAAGACTTTCCAAA
CTGGGAACATTCAGATTGCTCAGGGTATTGATAATGTTGCAGGACAAATTGTTAACAATGCCGATATCAACTTAAAGGTACAGGACAGTATTAA
AAATAATGCTGGTGAAATTCAGCTACCTGAATTGCAATTTAGTGGACAAAACTTCGAAAACCAACAAGGTAAATTCACGGCTAAGGTGGTGAAT
ATCAATGCACAAAATATCGATAACCAAAAAGGTGTGATGAGTGCTAGTCAAAGCTTTGATTTAACTGCCCAACAACTCAATAATAATCAAGGCC
GTTTACAAAGTACAAAGGCACTGAATATCACGGCAACTGAGATTGATAATCGCCAAGGTCAAATACTCGCTGCTGATGCACTGACGCTAAATTC
ATCCAATACCAATAACACACAAGGTGTCATTGCTTCAGTGAACGCGGATGCACAACTGAATATTAAATCTCTAGACAATAGCAAAGGGGAAATA
TCTGCACAGAATGTGCATCTGACAGGTCAGAGCTTAAAGAATCAACAAGGTACGATTCAAGCCAAAACAGCAGACTTGAATATCAATGTTGATC
AGATTAATAATGGATCAACCAAAGATTTAGCAGGTAATCTGATTGCTGCACAAAATTTAAAACTGAATGCGCAGCAGCTACAAAGTACGGGACA
AATCTATGCAGGAGATACAGCAGACCTTACCGTTGAACAATTAAAACAACATGGTCAACTTGCAGCACTTAACGCAGTTCGTGTTCAATCCAAA
GATATTGAGAGTGACCAAAATGCAATTTGGGTAGCAGGACTTGATCAAGAAGGCAAGTTGAGTAATACCACGTCTGAACTCAATATTGATGCAC
AAAATGCACAGATTGCGGGTAAAATTTTATCTGGAGATCAGATAAACATTAAAGCTGCTCAAACAACTGACTTAAGTCAAAGTGAAAGCCAAGC
CAAAAACGTTCAAATTGAAACGACACAATTCGATACCAGCAGTGCCAAAATCATTGCAGACCGACAATTAGATATCCGTGCTCTACAAAGTATA
AATAATGAAAAAGGGCAATATAGTGCGGAACAGGTCAATCTCAATACAGCACAACTCAATAACAACCAAGGTTTAATCCAACACACAGGCAAAA
ATGATTTTATTCTGAATGTGGCAGATCGTATTGCCAACCATGCTGGGAAAATTATCAGCAATGCCAAAACGACTGAAATTAAAACCAATACATT
AAGTTCTGTAGCAGGTGAAATTTTACATGCAGGTGATCAGCATTTAAAAATTACTGCGCAAAATTTGCAAGGACAACAAGGCAAAATTCAAAGT
AACAGTGACTTACAGCTGAATCTAGGCACTGCCAATTTAGACAAAGCTTTGACTGCAGCACAAAGTATAAATCTGACCGCAACAGAACTGAGCC
ATCAACAAGGGCAATTGATTCAAAATGATGCCAATGGGCATTTGCAGGTTAACGTTGTACAAACCTTAAATAATATGTCAGGTGTGATCAGCGC
AGCGGGTAATGCAGACATTAAAACAGCGGATTTAAATAATCAAAGCGGTGTGATTCAAACCCTCGCAAATAAAGACTTGAGCATCGAAAGTCAA
AAACTGGAGAATCAGTCTGGTAAAATTGTTGCAGGGCGTGATGCAGCTATAAAAACGACTCAATTGAATAACGACACAGGGACTGTCTACGCAG
CAGGAAAATTGGGACTGCACGCAACACAAGATGTAAGCAACCAACAGGGTTTAATTGCTGCAAAACAATCACTCAACATTGAAGCACAGAACCT
AAACAATTCTAAAGGGCAGATTCAGACCGAATCTGGTGATGCAAATCTAACCATTGCCCAGATATTGAATAATCAAGCAGGTCATATTCAAGCT
GCAAACGCATTGAATATTGTGGCTACTCAAGTTGAAAACCAAGCCGGACAGCTGCTTTCAGGTACGGATACACAGCTTGATGTTGCACATCTGA
ATAATCAATCAGGCACAATCTACTCTAAAAAACAGCTGGATCTCAATGTTTCAGGCAGCACGGATAACAGTGCAGGTACCCTTGCGGCAGACCA
AAATCTAAACCTAAACACGCAGAACTTATTGAATCAAGCAGGACAAATCCGTTCAGAAAATGCTGATTTAAAGTTAAATATCGCTCAAGATGTT
CAAAACAATACAGGTTTAATTTCAGCCGCAAAAAATCTGAATCTGACAGCAAAAAACATTACTTCACAACAAGGTAAAGTCCAGTCGGGTGCAA
ATGCCAATATTCAACTGAACAACTTTGACAATACTGAAGGTGTGGTTTATGCCGAGGAACAATTACAGCTTTCGGCAACAGGTAAATTAAACAA
TACGCAAGGTATCGTTGCAGCAGAACAATCGACTGATATTAGAGCTGGCTCTGTCATCAATGATGCTGGACAAATCCGTTCACAACAAGATCAA
TTGAAACTGAACGTTCAGCAGGATATCAGTAACCAAAATGGTGAAATCTCTGCGGCAAAATCGATTGAGTTGAATGCACAAAAAGTCAGTAATC
AAAAAGGTAAAGTGATTGCAGGCGAATCACTGAATGTTACTGCTCAGCAAGTTGATAGTATTGAAGGCACAATCTACGCCAAAGAGCAGTTGCA
TCTTACAGTTGCAGATCAATTCAATAATCAGTCTGGCACGATTGCAGCCAACCAACTCGTTCAGATACAAGCTAAAAACCTGAATAATACGGCT
GGAAAAATCCGCTCAGAACAGAATCAACTGGATTTGAATGTAGAGCAGAACCTTGACAATCAGACAGGTCAAATTTACGCAGGTAGCCAAGCTA
ACATTCACACAACTGAACTGAATAACCAACAAGGCACCATCTACAGCCAAAATCAGTTGGACTTAAATGCCTCTCAACTGAACAATCAGCAAGG
GCAGATCTATTCAGGAGGTCAAGAACAGCTCAAAGTTCAGGGCGATATTCAAAACCAAAAGGGTGTATTGGCAGCTGGACAAAACTTAAACATT
AATAGTGCAGGGTTGGATAATACAGCAGGAACAATACGCTCAGAAAATGCTGATATTACGCTGAATGCACAAGGACAGTTGATTAATGCTCAAG
GCGATATTTATGCTGGGCACAATGCAAGTTTAAATAGTGTTGGTTTGGACAACAGTTCTGGACAAATTGCAGCACAGAATCAACTCACTATTGA
TACGCAAAAACAGCAGCTCAGTAATCAAAATGCTAAAATTATCGCTAGGGCTGTAGATTTAAAAACTGGAAAACTGGATAACCAAACAGGCTTA
ATACAAGCTGAACAGTCAGTTAAAATCGATACTCAACAAAATGCCTTGCTCAATAATAACTCAGGAAACAATGCGGGAATACTCAGTCAAGGCG
GTTTAGATATTGCCAATGTTTCTCAACTTGAAAATAGCAATGGTTATATTGCAGCGATCGGTTCAGCCAACCTGACTGCTCAGAATATCAATAA
CAACAGTGGTCAAATCAATAGCCAAGCAGACCTAACCATACAACAGCAGTTGGCAGGTGGTCGTATCGATAACGTTGCTGGTCAAATTCAGGCA
CAAAAAAATATCAGTTTAAATGCCGACACGATTAATAACGCAGGCACAGTGAGTCACATTATTGCTGGAGAAAAGCTAACAGCGAATGCCAGCA
AAGTGATGAATGAGCAGACCAAAGATAGCAATTTACTCGGTGGTTTACATGCAAAAAATATTGAAATCAATGCGGGCGAGTTGGATAACCAATC
TGGCGTGATTCGTGCCAGTGAAAATGCAACATTGAATATCAACAACCAATTGAGCAATCAATTGGGTTCAATCACTAGTTTAAATCGTTTAAAT
ATTGGTACAGCAAATAAAATCCTAAATTTAAACAATACAGATGGTGAATTACTGGCAAAAAATCAGCTCAATCTGAAAGCCAATGAATTGGTTA
ATAAAGGCAAGATTATCAGTGAAGGTAATGTCGATATTGACTTGATGCAAAGTTATAGCCATACCCAAGCGGACCAAATTGCGGCGAATGGCAC
ACTTAAACTCAGTACCGAACAGGATTTAATCAATCAGTCTGAACTGAGCGCAGGACAAAAAGTTGAACTGAATGCAAAAAAATATTCAAAACCAA
ACAGGTGCGAGTATTTCATCTAATCAGACTCATCTGATTGCACAAGATACCATACATAACCAAGGCTTAATCAATGGTGAGCTCACCCATATTC
AAGCCAATCGTGTTTGGAATGATGGTGCACGTATTTATGGTACGCAAGTTGCGATTCAATCCAATACGCTTGATAACAAATCGAATGCTAATGG
CACAGGTGCAGTCATTGCTTCACGTGGAGATATGGATTTAGGGATTCAAATCCTAAATAACCAATCTGGTGGTGTGGTTAAAGAAAATGGACGC
GATAACGCTTGGATCTTTAGTGCAGGTCAACTCAATGTCGGTGGCAGCCTAAATGAAGAACTACAAGCACAAGGCAATGCCGACAAAATTTACA
ATGGCAGTGCCGTTATTGAATCTCTAGGAGATATGTATCTCGGTGCAAATAATATTCAAAATACAAATGAAAACTTGGTTATAGCGCTGATTGA
AAAATATCGAAAACAAGTACATGAATATGGCAAAGATGGCGAGGTTTGGGATTCCAGTGTAATTCGCCTAGGTTCATCATCGCGTGGCTTAAGT
AATGCCATACTGTATGTACCACAAGAAGAAGGTGGAGCGCCAACAAGAGAGATTGGTGAGGATTGGAGCTATTATGAATATACTCAAATTCACA
GTGAAGATGAGGTTCAATCCACAAGCCCCGCACAAATTATTGCGGGTGGCAATCTAAGTTTTACACCAGATGCTGACTTTGTAAATAAAGACAG
TCAAGTGTTGGTTGGCTTGCAAATCACCAATGGCATCGGCTCAATCAGCAATCAAAGTACCGCCTTAAGAAGTGTAGATCAGACCGCACCTGGG
GGATTCAGTCAATGGCATTCTGTCGGTTGGAATACCAAAGGGACGGAGCATCGTCATCGATGGGGAAGTAAAGTCAATTACCAACCAGCCGATA
TCATCAATACCATGCCGATCAGTTTAGGTATTGTAAAAGAGTATACTTCACAGCAATCTAGCAATCCTGTTGAAAATTTAAATAGCACGAATGT
TCAAACTGAGATAGCACAGGCACAATCAGTTGATCTAACCGCATTAAGTACACAAAAACTCAATAATCAACAACTCGATTTAAATGCGGGTCAA

AGCATCGCTGAAATTGGAAAAAATATTCAACAACCTGATTTGCCTGATCAAATCGGCGTTGAAGCCAATCCGACCACAGACATACAAGCTGAAC
AAAACAATATTGATCGCGTTGAAGGCGAAGAATTAAACCAAGTGAATAACACTGCTTCAGCAGTCGATTCAGCGACCGATATTACAGTTCGTAC
GGTATCGCAAGACTTTTTAAACTTACCGAGTAATGCTCTATTTATCACCACCAAAGACAGTCAAGCACAATATTTGGTTGAAACTGACCCAGCC
TATGCAAATTATCGGAACTGGTTGTCTTCAGACTACATGATTAAAGCATTGGGTCTCGATCCTGCAATGCAACAAAAGCGCATCGGTGATGGTT
ATTACGAACAGCGTCTTGTACAAGATCAAGTTGCAAAACTCACAGGTTATCGTTTCCTTGAAGGCTATGGTTCGGATGAAGAACAATATAAGGC
ATTGATGAACAATGGCTTGTCATTTGCTAAACAATACAAACTCAGACCGGGTATTGCATTAACAGAGACGCAAATCGCTCAATTGACCAGTGAT
ATTGTTTGGTTAGAAGAAAAAACAGTCAAACTTGCAGATGGTACGACCACACAAGCACTCGTACCTCAAATCTATGTCAAAGCAAGAGTCGGTG
ATTTAAAAGGAGACGGTACACTTATTTCAGCGGATTCAATTCAATTGGATGTACAGGGTGACGTATTTAATTCAGGTACCATTGCAGGACGACA
AGCCGTTGTTTTAAATGCTGAGAATGTCGAAATACTGAATGGACGAATTCAAGCGAATCAAGTTGGGCTCAATACAAGCAAAGATTTAAATATT
ATCGGTGGACAAATTCAAGCTGAACAAGCTGTTGATTTAAATGTAGGGCGAAACTTTAATTTAGAAAGTTCAACACAGCACAGTGAAAATAAGA
TCGGAGAAAGCACCTTTACTTATACAGGTCTTGATCGTGTTGCAGGTGTTTATACCAAAGCACCAACAGCGATTAAATCGAGTGATACTGAAAA
CCTAAAAACCTCGATTTCAATTCGTGTTGGTGGTGATTCGGATTTAAAAGCAGCTCAAATTCAGAATGCAAATGGCTCTACTCAAATTCTGACA
CAAGGTAATTTGAATATTGGTGCCATTCAAACAGGCATTAGCAATAAAGGCTATGCCAATGAAAAGAACTATAACTATTCAATCACTCAAATCG
ATCAAGGTAGTTCAATTCAAAGTCAAGGAACAGCTAATTTAAGCGCAAATAAAATTCAAGGTAAAGCAGTCGATATCGCAAGTCAGCAAGGTGA
TATTGTACTCAATGCTCAACAAGGGATAAGTCTTGAAAATGGTAAAAACTTACATAGCACTGATAGTGCTATGTCAGCACAGTCAAGTGGGATG
CTTGGGCGCAATACTGAGCGTAATGAGGTTTGGAAGTCAGATCAGTCGATTGCCAATAATTTAAAAGCTGGTGGCAATATTGTGCTGAATACAT
CGCAAGGTGATATCACAGCGACCCATCTAAAAGCGAATGCAGGAGAGACTATTCAGGTTCAAGCCAAAAATGGTCATGTAACCCTAAATTCAGC
ATTAAATGAAACCAGTCAAAGTACGACTTCTTCAAGCACAAACTTTGCAACTTATAACAATCGACAATCTGGTTATATCGATCAAGAGGTTGCG
CAAACCCAGTTGGTTGCTGGTAAGAATGTAGATATTAATGCTGCTAAGAATATAGAGCTACAGGCCAATGATGTACAGGCAGGAAACAGCATCT
ATGTGGGCAATACTTTGATGCAACGTCAAGTGGATGGAACATTAAAAGCTGCTGATGGCTCCGTGATGCCTGAAAATGTAAAACTGAGTACATT
GGAAACACATGACCAGCAATGGGACGAACAACAAAAAGGTTATCGTGGTGTTGTTAAAGAGTTAATGAAAGTCACCGAAGTTGGTTTAGCTGGC
GTTCAATCTTTATTCCCAGGTGTTAAAGTGGACACTAAACTCACTATAGGTGAGTCCAACAGTAAACGCGAAGAGCAAATTAAACAAACGGGAA
CAAATTTAACAGCGAACGATGTGTATGTTGGATCATCAGGTCAAACGACACTGACCAGTTCTGATATTACTGCAAAGAATACGATTCTTTCAGG
TCAAAAGGTGACATTGAATGCGGCTGAAGAACAAAACATTTCAGCCACTTCACACAGTAAGGAAACCATTGAAGGCTTAGGCGTTAAGCTCAAC
AAAGATAGTGTACGTTTAGGTGGCTTTGTGTCAGAAGATACCACGCAAAGTACTAAAACCACCGAAACTACTCACAAGGCAGGTTCAATCCAGA
CTGAAAATCTTAAAATACAAGGTGCTGAAGGGGTGGATATACTCGGTCAAAATATCAAAGCGACAGGTGATACTGTTCTTGATCATGGTCGAGG
CGATTTAAATATCGGGGGTTATGAAAATAAAACCACGGTTGAAGATAAGACACATAAAGAAACGATCAGTACTGAAGTGGGTGTACGAAATGCC
TATTTGGATGCTGCACTTGCAGTAGGTGCGGTCAAAGATGCAGCAGAGGTGGTTAAACAAGCCAAAGATCAATATAGTCAGGCACAGCGTGATT
ATGCATCAGGTAAAATCACCAAAGAAGCACTGGACGACACCAAAGCCAATGTTGCAATGGCAACAGCCAATCTTGCCAATGCACAAATTGCGGT
AGGTTCGGCAGCAGCAACCGCAGCAGCAAGTTCAGCAACTTACGGCTTTACCATTGGTGCAAACGGTGAACGTATCGAAACCACCACCACGACA
AATACCACTCAAGGTAAATGGCAAGGCAGTCAGTTAGATTTAAATAATTTGACACTTAAATCAGAAAATCAAGATGTTAATTTGCAAGGTAGTC
GCTTAACCGCAACTGGAACGACCACATTTGATGGAACGAAAGATTTTAATATCAGTGCAGGAAAAGAACACGGTCAACAAGAGACCAGCAGTAA
AACCAACAACCAAAGTGTGAGCTATACCTATGGCGGTGGTGGCAGTGCATCAGTGGGCAAGCAAACATCGAAATCACACGCTGAAAGCCTAACT
CATGTGAACAGTGAGGTTGCATTAAACCGTACAGAAGGTCAGTTGAATAAACTCAATATTCAGGGCGGTGAAGTTTCTATTGCTGATCGGGGTA
ACTTACAGGTTAATCAAATCCATGTAGAAAGTTTACAAGATACAGCATCAGGCAACAGTAGCAGCAAAGGCGGTTCAGTTGGTGTAGGCATTGG
TTCAGGTGGTTTGAGTAATGTCACAGCAAGTTATAACCAAGCCAAAGGTCAAAATGATAAAGCTTGGGTGAATGAAACCAGTAAATTGCTGATC
GGCAATGCACAAAATGATGCAGACCTAGATGCGATGGGTGTGCAAAAGGTGAGTAATATTGGTGGCGTGATTGCCAATTCAACTAAGAACTCAG
ACGCAAGTCTAACAGATCATGGCAAACTGAATTATACAGGAGCACTCGAACTTAAAGATTTACAAGATCATAGCTCAGAAAGCAACCGTGGCTT
TAATGTATCGACCAGTGTGGGCACATCGATTAAAGGTGAGAGCAAGGAGAGCTCATTCCATCCAAGTGGTAGTACAACGGTTGGCTTGCAAAGT
ACAGGGAATGAACAAGAGCAGTTAACTAAAGCGACAATGGGTCAAGGCACATTGAAAAATAGCACTGCGTTGTCTAACCGTGATATTAACAATA
CTCAAGAAATCACCCGAGACCAAGCGACAGGTTTATTGAATGGTTCAGTGACTGTCGACAATCGTTTATTGACAGAGAGTGGTCGTCAGCAGAT
TATTCAAGAGCAGAAGGATTTACCAGAGAATTTAAAGCAAACAACACAACAAATAAAAAATGATGTTGAAAAATTAGCTTTAAAACTACCTGAT
TCGAAAATTCAAAATAAAATAATCACTCTCCTTAACCAAATGGTCGGAGGAGATATGATTGCTGATGCATACAAAGAGTTCTTAAAGAATGGAG
GATCAAAAGAAGAGTTTTTTGTTGTAGCGAATAATTCGTATGCACAGAATTATATTAAGGATTTAGCTGATGCAAATGAAAAAATACAAGAATT
GGAGTCTAAGGGATATAGTTTAGAACAAATATTGAATACGAGCATTGCTTCAATTGGTGATACTAATGTGATTTCAGTAGAAGGTCAGCATAAG
GTTAATTTAACAACTGATACCACGATTGGTATGCAATTGTTAACTGCATCGTATGGATTATCGAAAGGCTATTCTGATTTTCAACAGGAAATGA
ATGATAAATATGGAGTAGAGATTGATCCCGAAACACTTTCTGTTGTAATTGGAACGATACTTACAGGTCCTGCTAAGACTGTTGCAAATGTCTT
ACTGTCTAGTCAATATGGGGAATATATTCAAAAAGCCAAAGATAAAAGTATCGACCTTCTTTCAACAAGCATAACGGCAATATCGTATGGTACA
GATACAAATGTTATTTCTACGTATACGAATCCAGAGTTAAGGGATCAAATAAGTAAAGAGTCTATTAAAACACAAGAATGGTCCAAGGATGTAA
GTGAGAGTAAGGAAGGAGCAAAATTTTTAACAAACATCATTGCAGGGATTGTAGTTGGTGGGGTAGGAAAGGGTGTTGTTAAAGGGAAGGACTC
TGAATATGACATATCTAAGTCAGGGTTTGAAATAGGTAGCACTAATCCTATAACAGGTCATAAAGTTTTAGGTACCCATAAAGATGAAAGCAAT
AATACAATTGGAGATTATATTGTAGAGGGAACAAAGCCTTCTACTAAAGATAAAGTAATAAAAACGGATATTCAATATTATGAAAATCCTGGCC
ACCATGACCCTAAAGGAGGAAATCAAATTGGTTACAATAAGACAAAATCAGTTCTGCCAGATAACCATAAGACTTTATGGGACAATAGTATTGT
AGCTTCTGATGGGAATAGGTGGGCAGTAGAGGTGATAAACGGGAAAAAAGTTTATCATCGCTTCCAAAATGATGGTAATGGTAATTTCCATTGG
AACGGTTCTACGAATGGTAAAACAGCAAGTGGGGTTGATAGAAGTGATGTTAGCAAAATTCCACCTGAAATATTGAGGAAGTAA*aatgttattt*
*ggtgatcctaggtattgatgttaatagtaagcaaagaaaagtc*ATGATTAACTTAGTG
